# Supplementary material for: Tetrahydroquinoline-Isoxazole/Isoxazoline Hybrid Compounds as Potential Cholinesterases Inhibitors: Synthesis, Enzyme Inhibition Assays, and Molecular Modeling Studies
Source: Int J Mol Sci. 2019 Dec 18;21(1):5. doi: 10.3390/ijms21010005 (PMC6981637; doi:10.3390/ijms21010005)
Supplement: Supplementary file 1 [file ijms-21-00005-s001.pdf]

Supporting Information

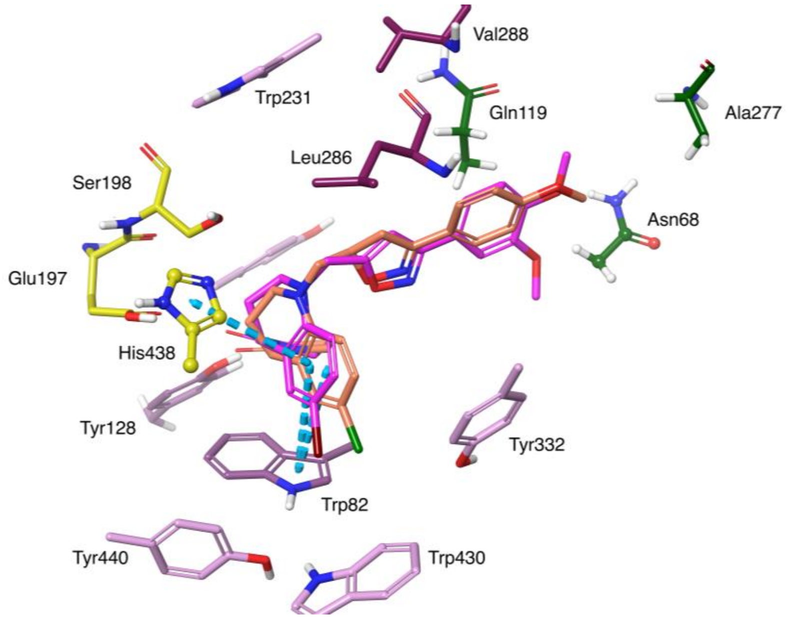

Figure S1. Predicted binding modes for compounds **5n** and **6aa** within the BChE active site. Compounds are shown in stick representation with magenta and fade-red-orange carbons for **6aa** and **5n**, respectively. Relevant amino acids are shown in the following coloring code: catalytic triad in yellow, anionic site in dark blue, acyl pocket in maroon, wall of gorge in plum, and peripheral anionic site in dark green. π-π stacking interactions are represented as blue dashed lines.

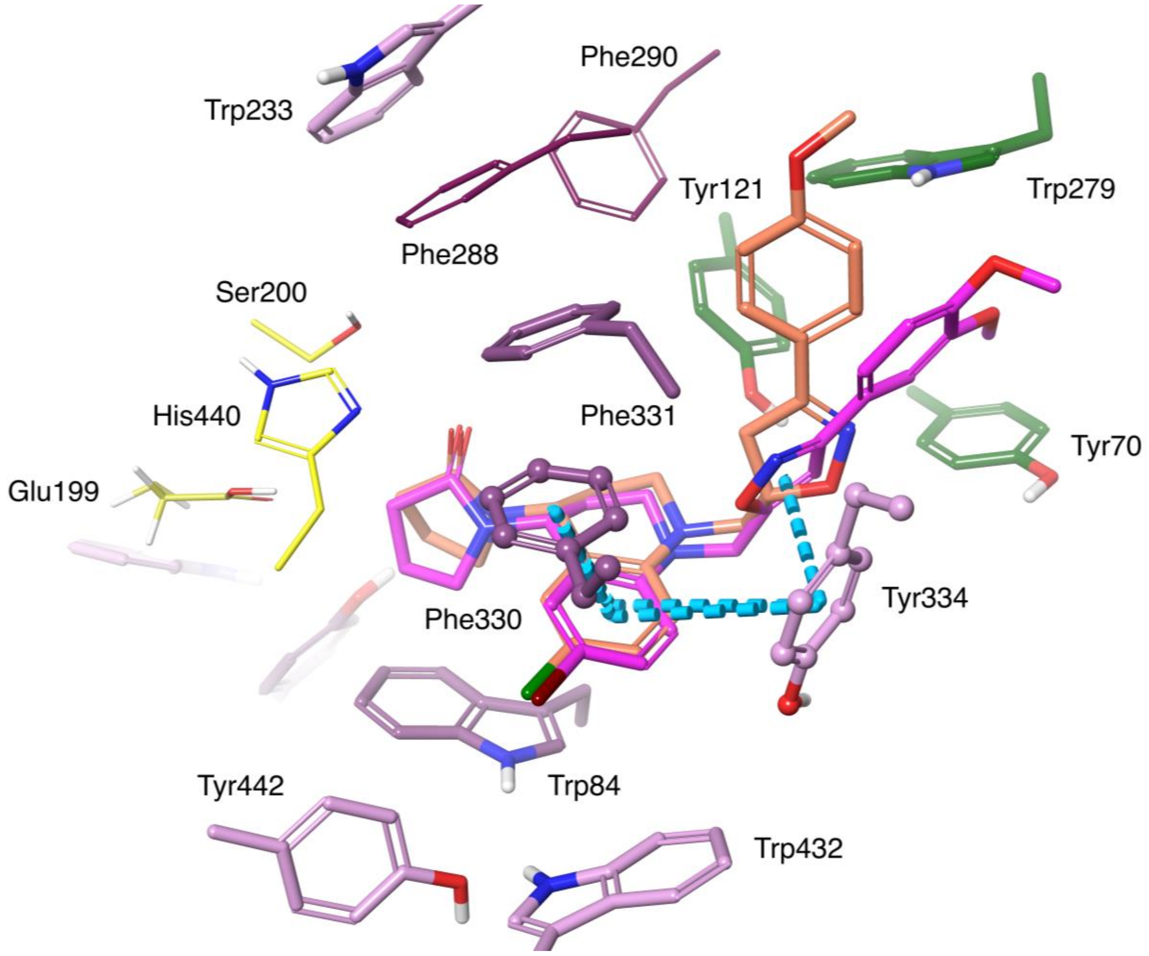

Figure S2. Predicted binding modes for compounds **5n** and **6aa** within the AChE active site. Compounds are shown in stick representation with magenta and fade-red-orange carbons for **6aa** and **5n**, respectively. Relevant amino acids are shown in the following coloring code: catalytic triad in yellow, anionic site in dark blue, acyl pocket in maroon, wall of gorge in plum, and peripheral anionic site in dark green. π-π stacking interactions are represented as blue dashed lines.

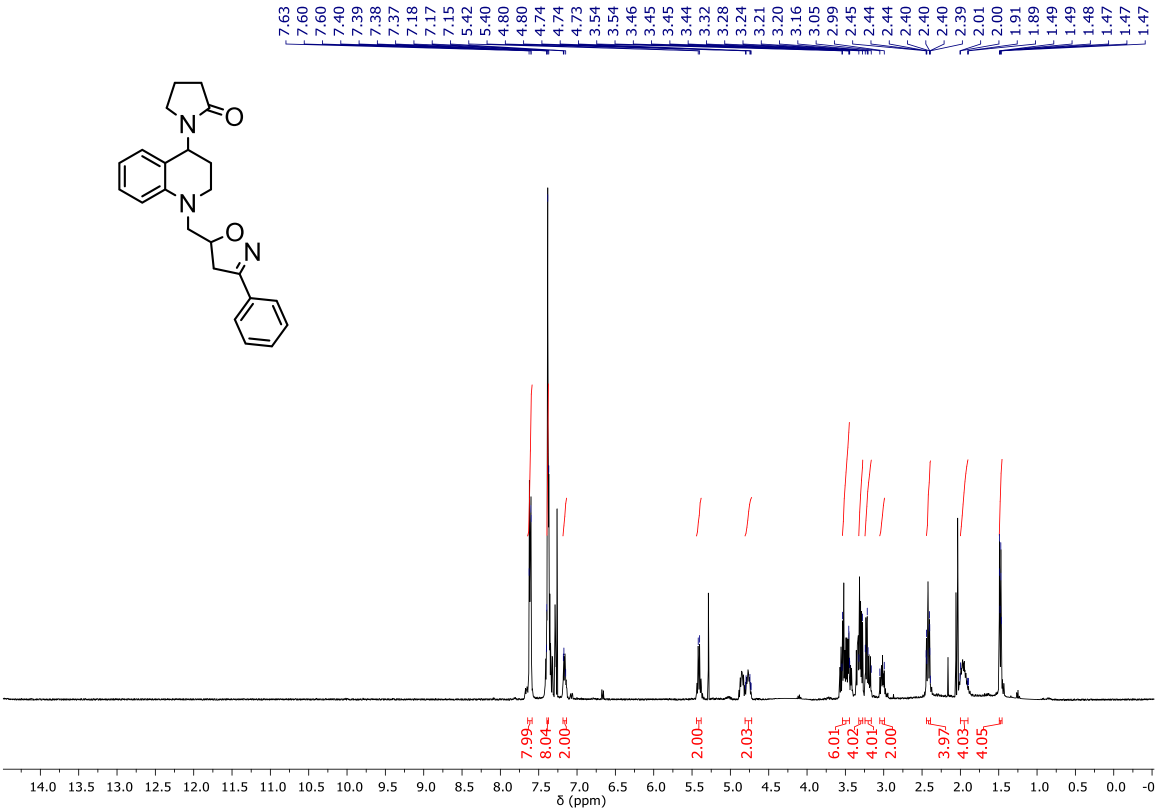

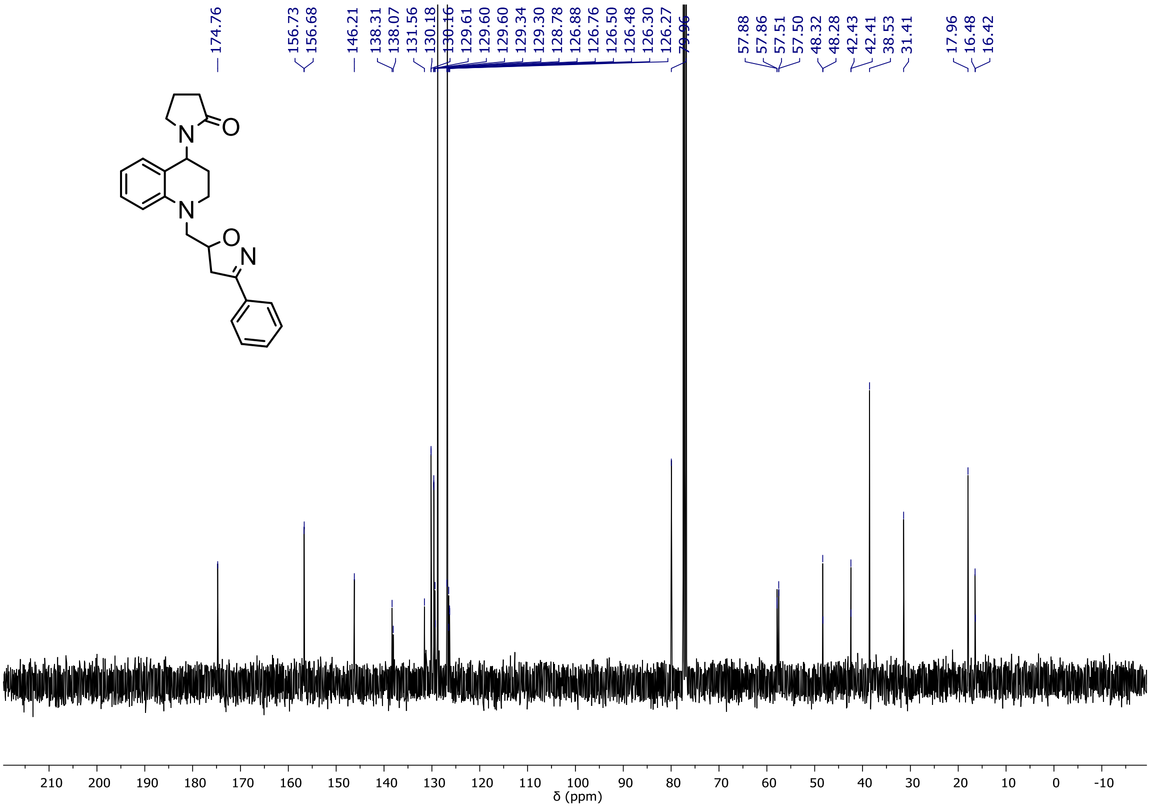

Figure S3. ^{1}H and ^{13}C-NMR spectra of 3-phenyl-5-[(4-(2′-oxopyrrolidin-1′-yl)-3,4-dihydroquinolin-1(2H)-yl)-methyl]-4,5 dihydroisoxazol **(5a).**

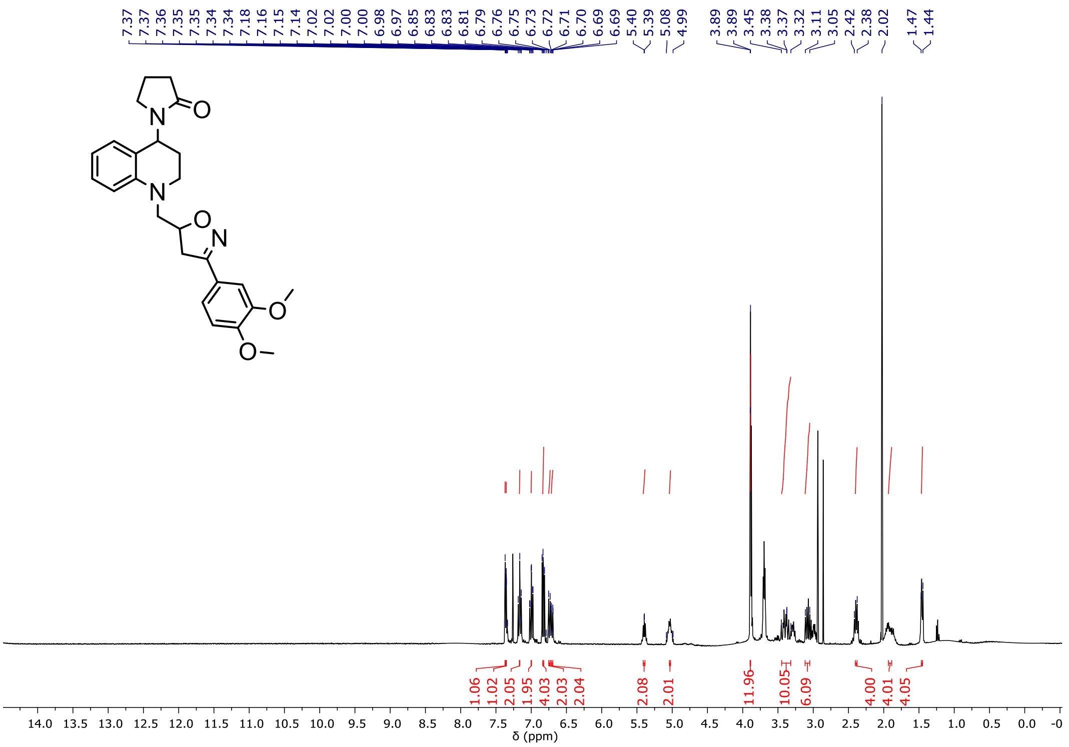

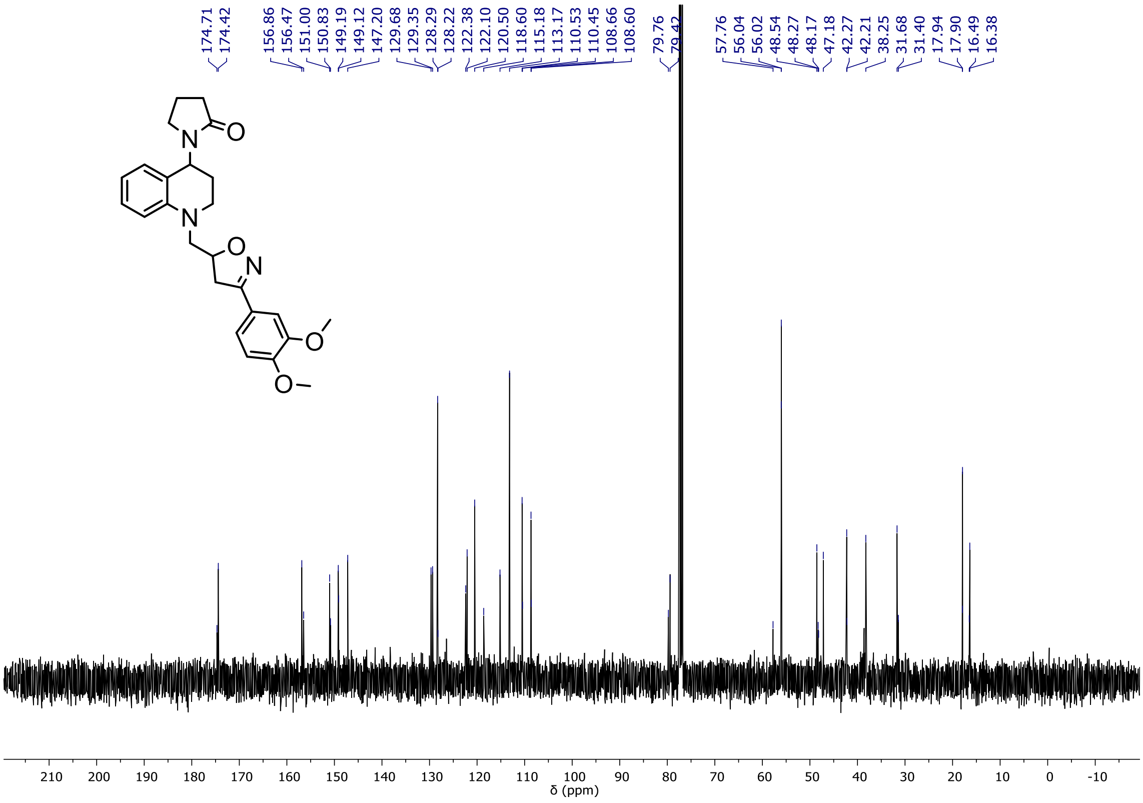

Figure S4. ^{1}H and ^{13}C-NMR spectra of 3-(3,4-dimethoxyphenyl)-5-[4-((2′-oxopyrrolidin-1′-yl)-3,4-dihydroquinolin-1(2H)-yl)-methyl]-4,5-dihydroisoxazol **(5c).**

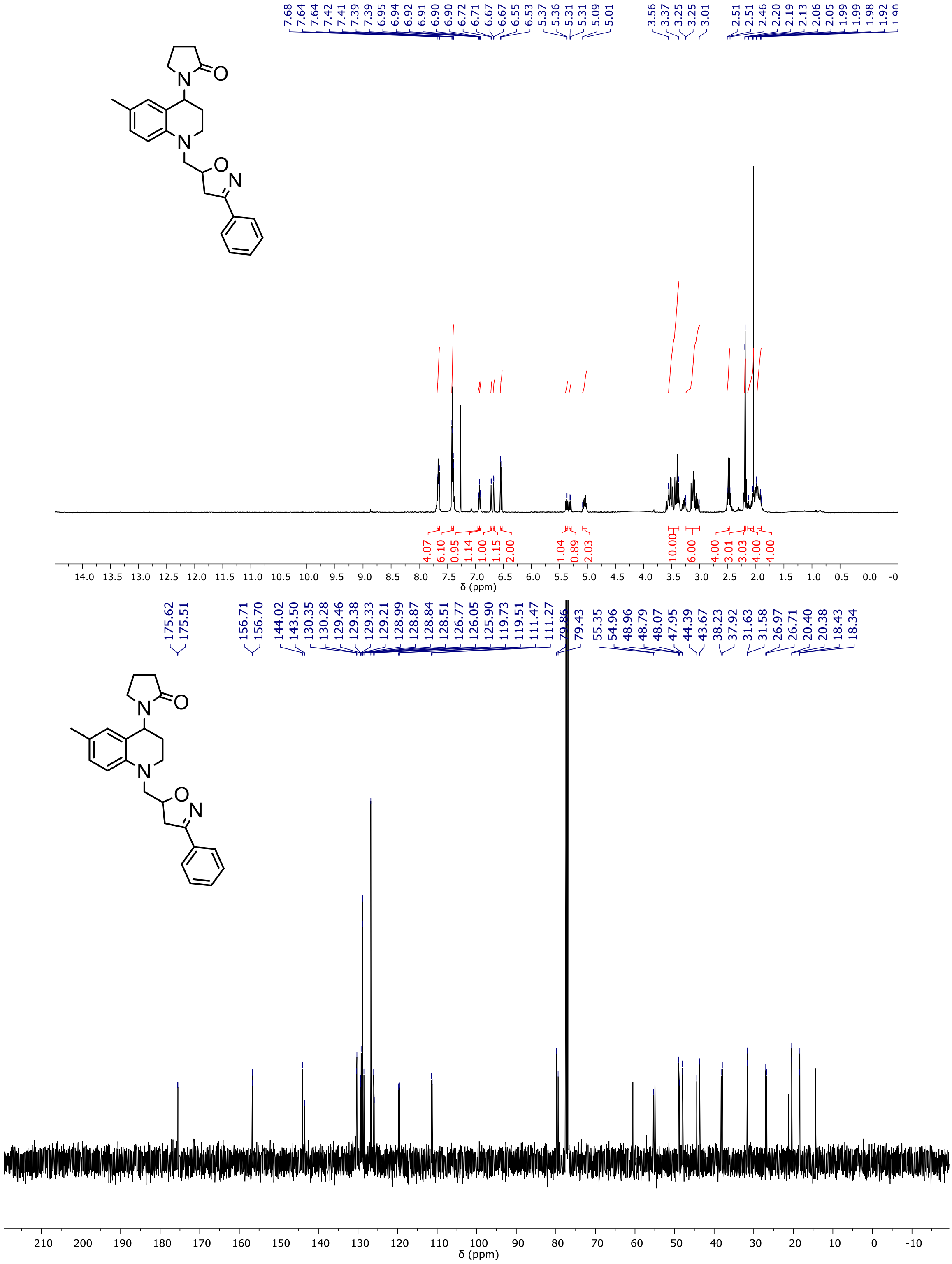

Figure S5. ^{1}H and ^{13}C-NMR spectra of 3-phenyl-5-[(6-methyl-4-(2′-oxopyrrolidin-1′-yl)-3,4-dihydroquinolin-1(2H)-yl)-methyl]-4,5-dihydroisoxazol **(5e).**

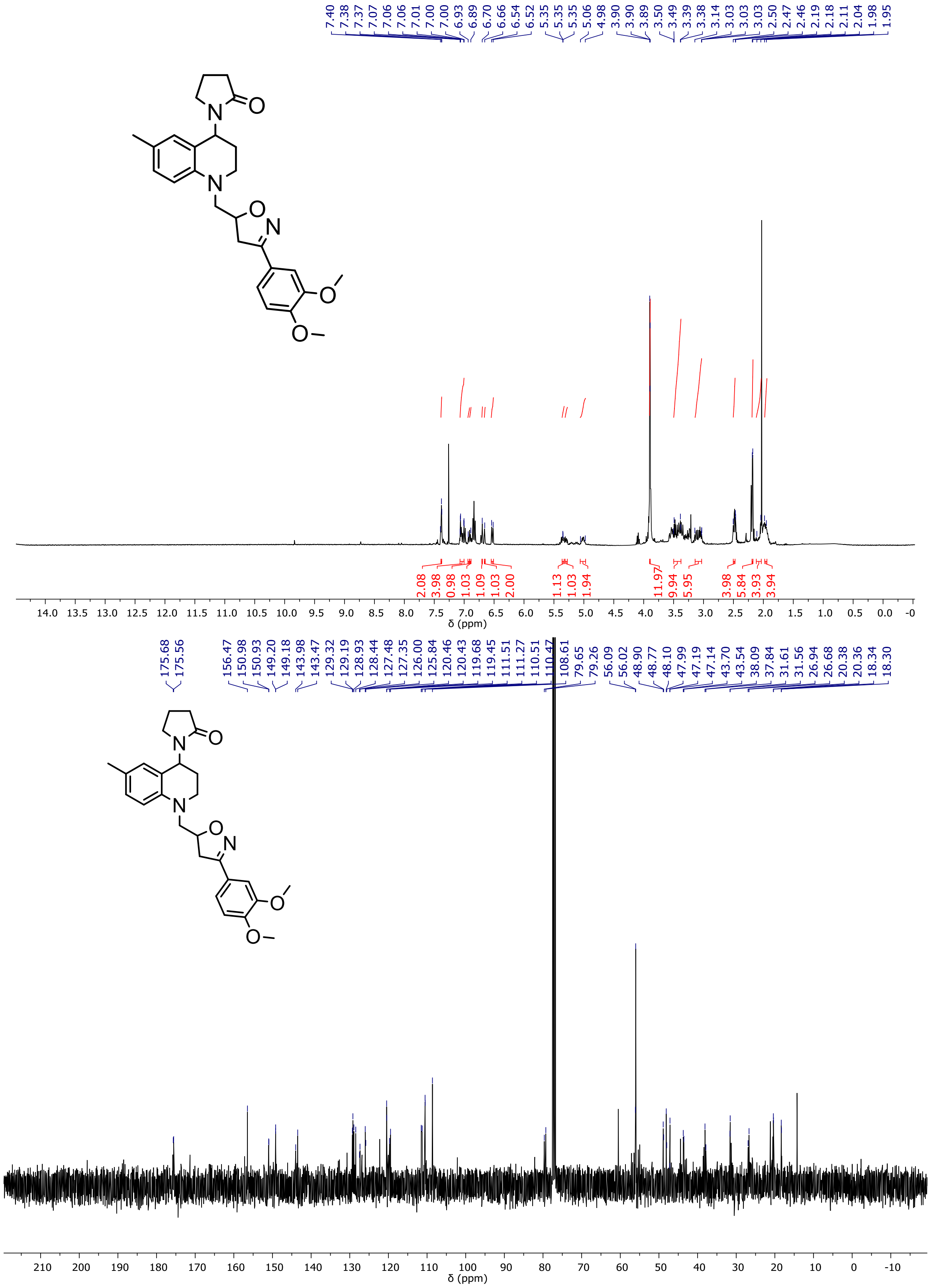

Figure S6. ^{1}H and ^{13}C-NMR spectra of 3-(3,4-dimethoxyphenyl)-5-[(6-methyl-4-(2′-oxopyrrolidin-1′-yl)-3,4-dihydroquinolin-1(2H)-yl)-methyl]-4,5-dihydroisoxazol **(5g).**

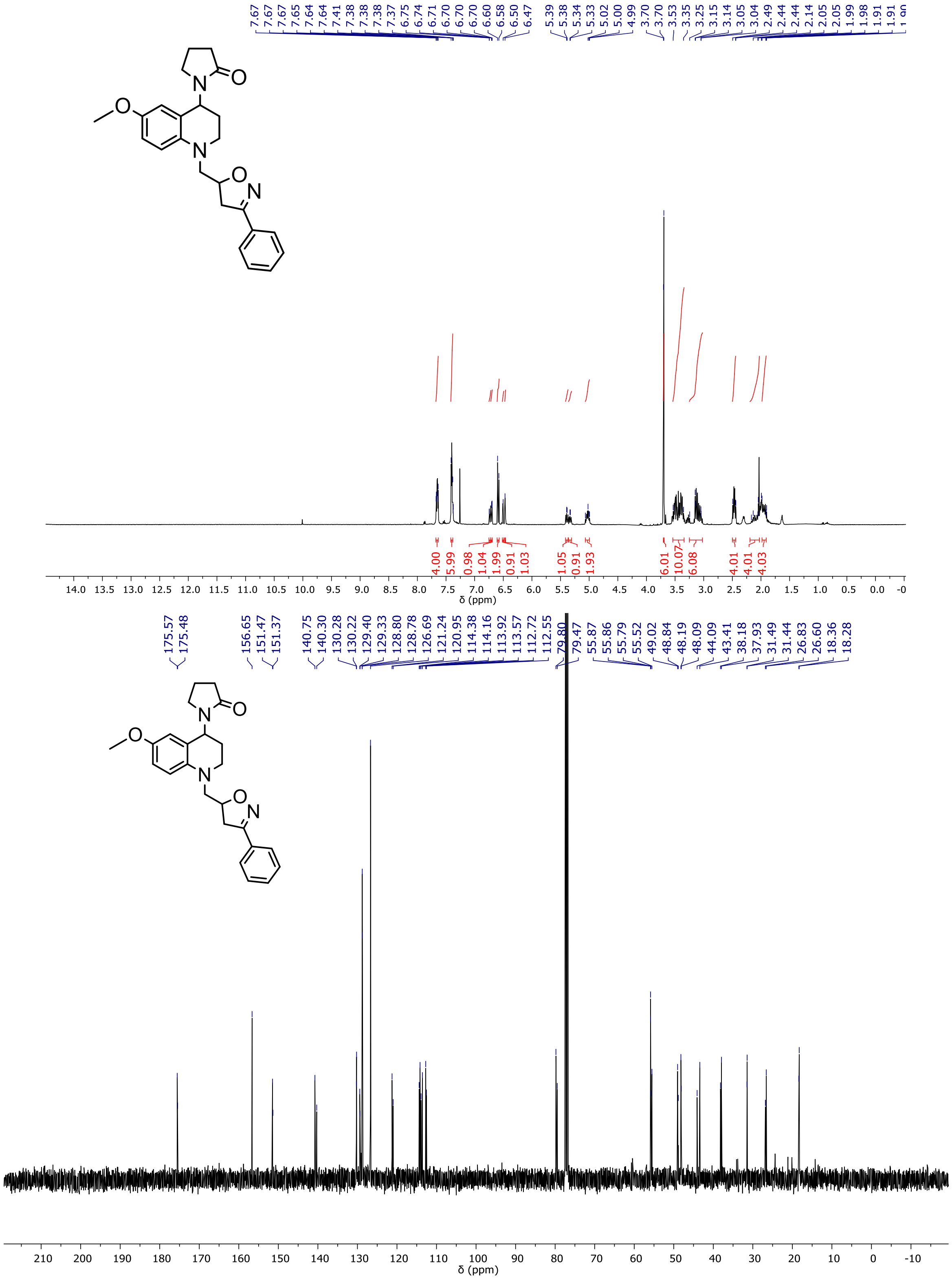

Figure S7. ^{1}H and ^{13}C-NMR spectra of 3-phenyl-5-[(6-methoxy-4-(2′-oxopyrrolidin-1′-yl)-3,4-dihydroquinolin-1(2H)-yl)-methyl]-4,5-dihydroisoxazol **(5i).**

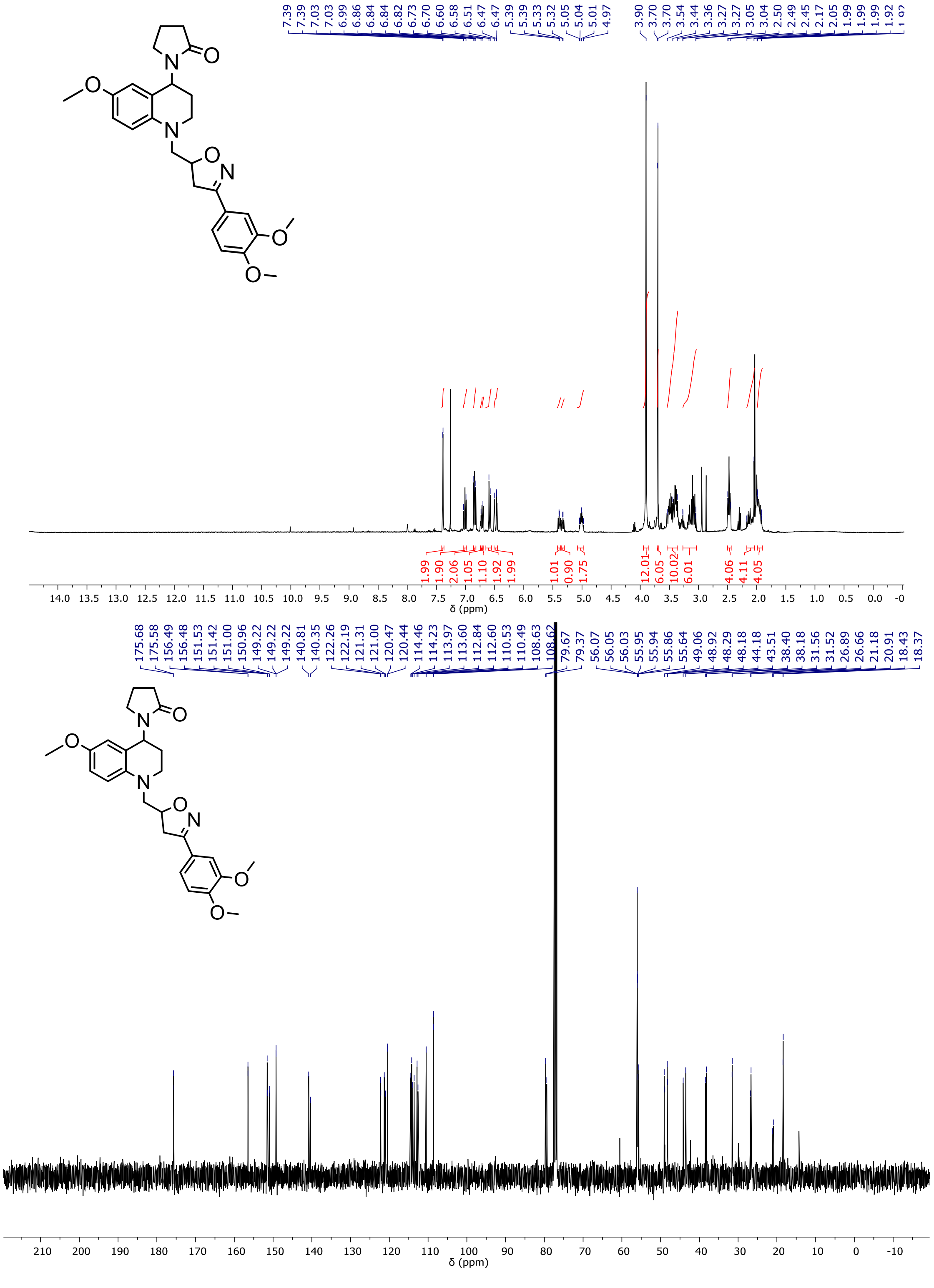

Figure S8. ^{1}H and ^{13}C-NMR spectra of 3-(3,4-dimethoxyphenyl)-5-[(6-methoxy-4-(2′-oxopyrrolidin-1′-yl)-3,4-dihydroquinolin-1(2H)-yl)-methyl]-4,5-dihydroisoxazol **(5k).**

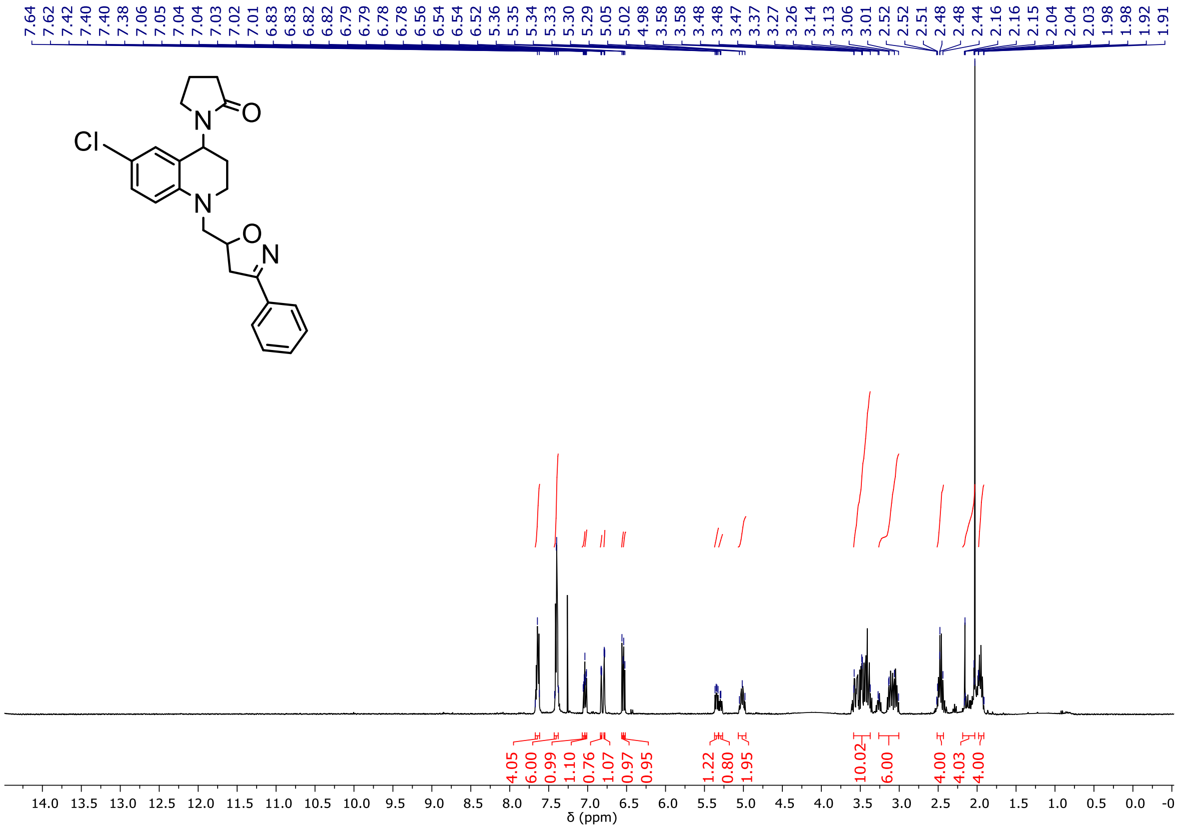

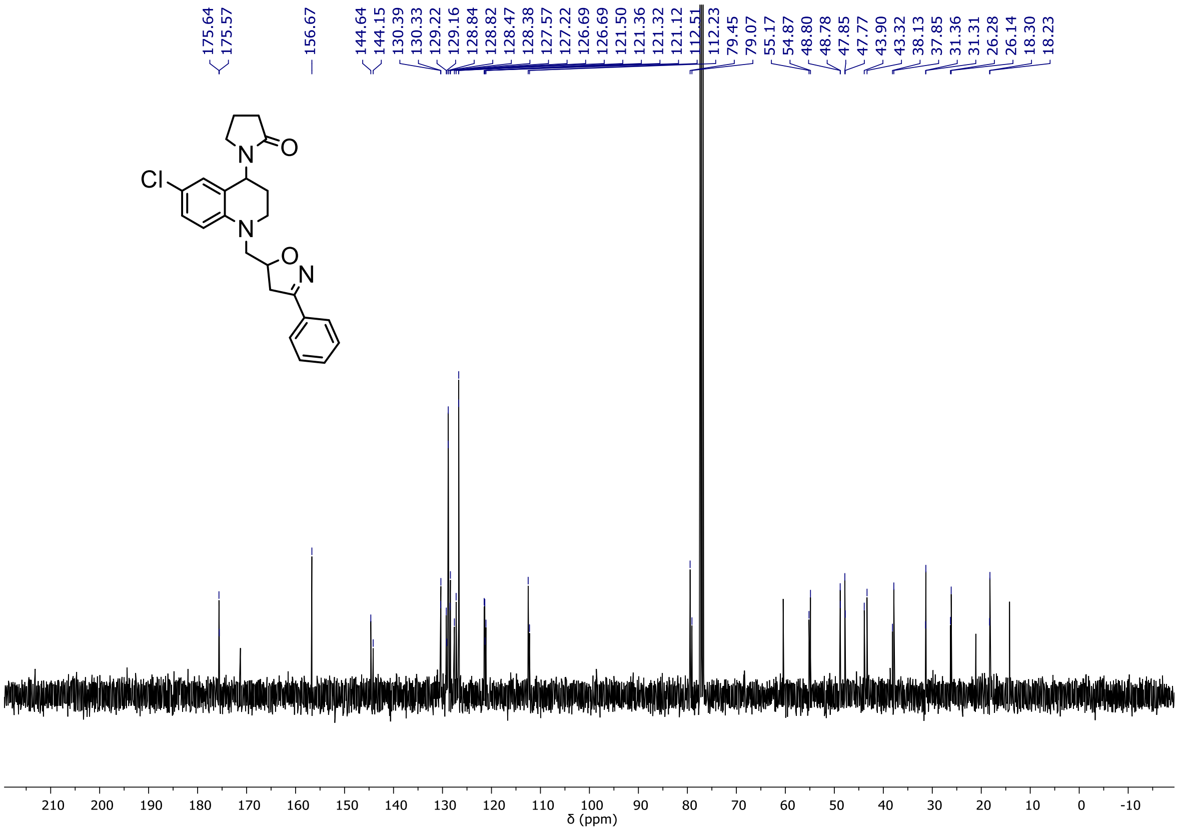

Figure S9. ^{1}H and ^{13}C-NMR spectra of 3-phenyl-5-[(6-chloro-4-(2′-oxopyrrolidin-1′-yl)-3,4-dihydroquinolin-1(2H)-yl)-methyl]-4,5-dihydroisoxazol **(5m).**

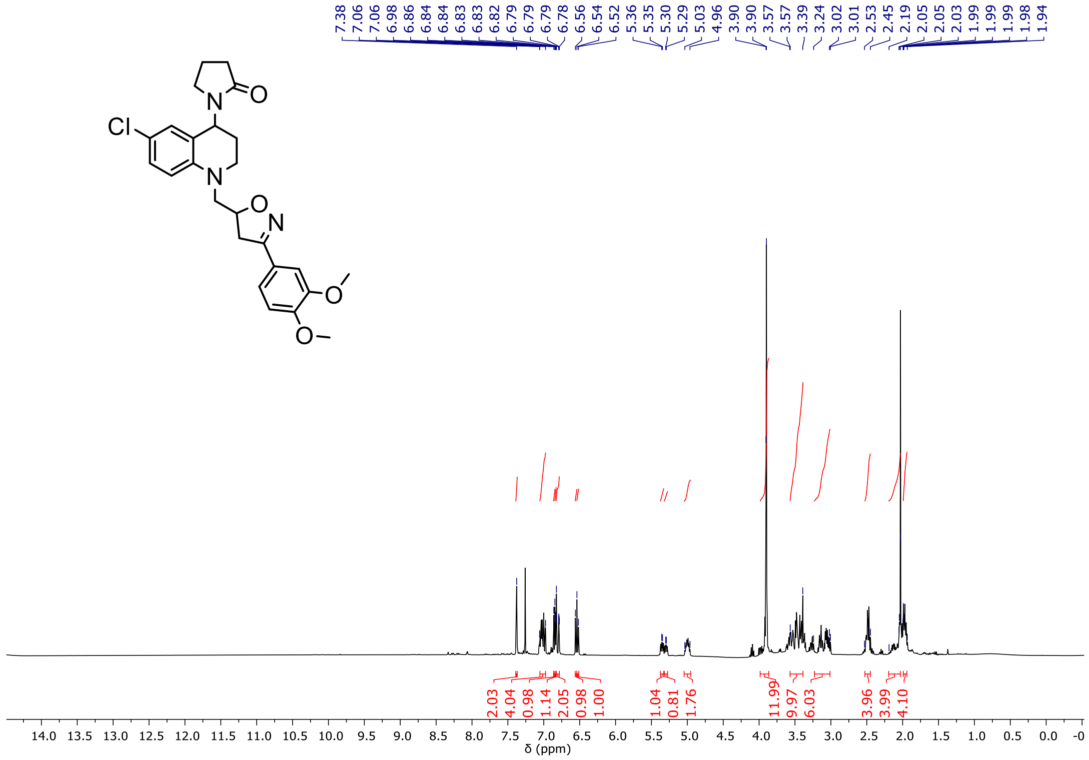

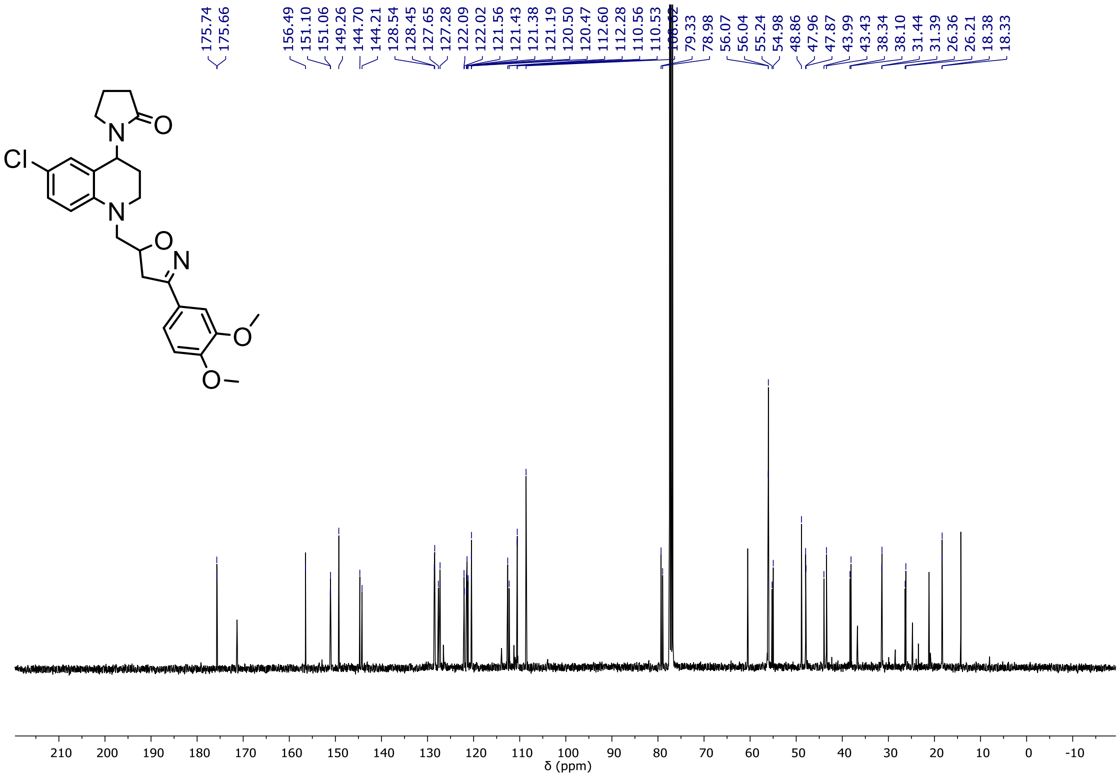

Figure S10. ^{1}H and ^{13}C-NMR spectra of 3-(4,5-dimethoxyphenyl)-5-[(6-chloro-4-(2′-oxopyrrolidin-1′-yl)-3,4-dihydroquinolin-1(2H)-yl)-methyl]-4,5-dihydroisoxazol **(5o).**

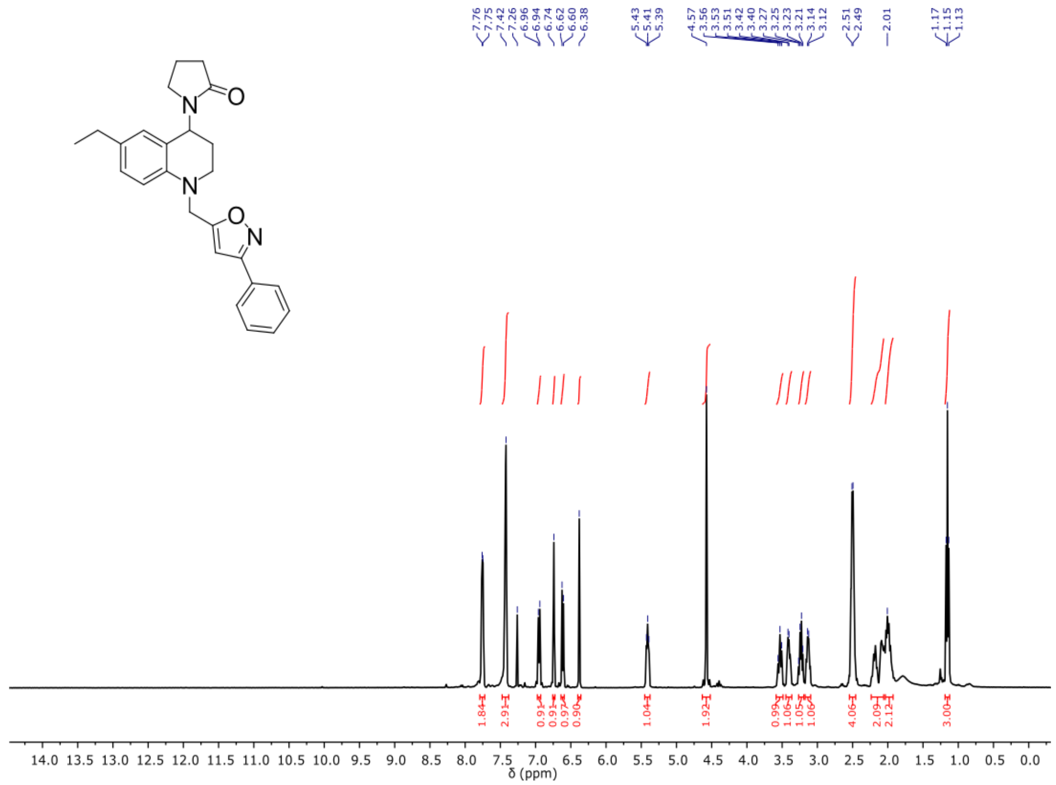

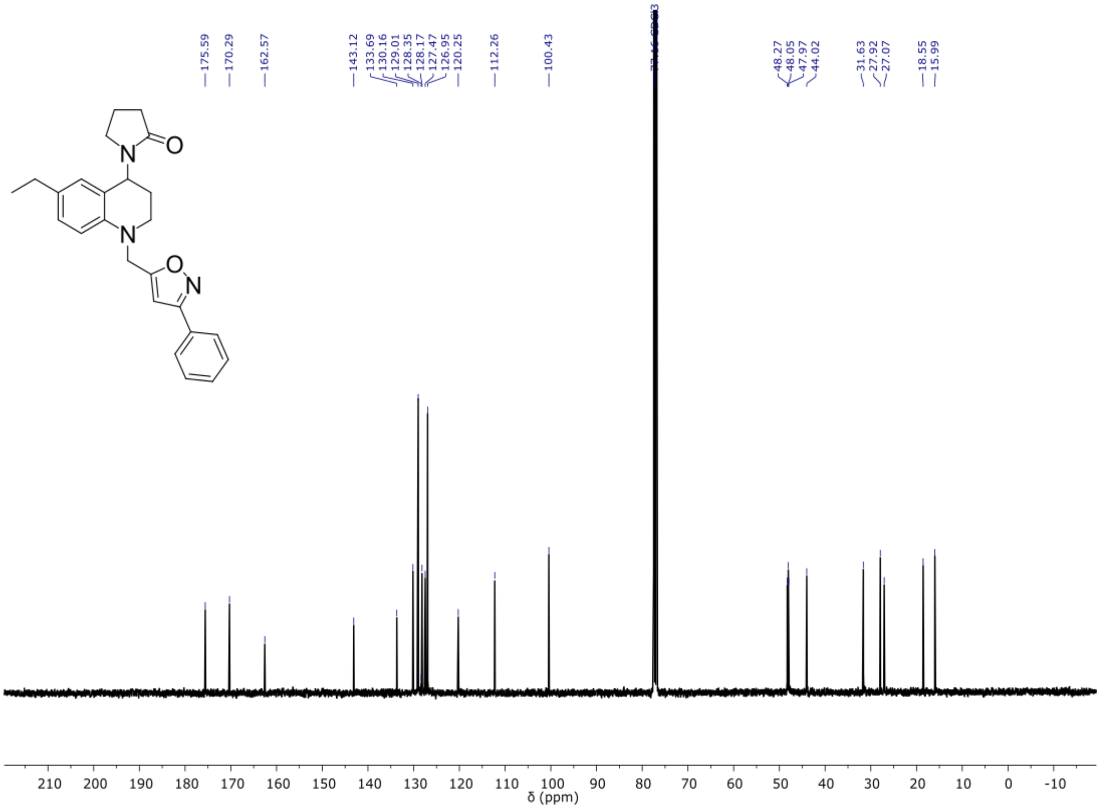

Figure S11. ^{1}H and ^{13}C-NMR spectra of 3-(phenyl)-5-((6′-ethyl-4′-(2′′-oxopyrrolidin-1′′-yl)-3′,4′-dihydroquinolin-1′(2′H)-yl)methyl)isoxazol **(6q).**

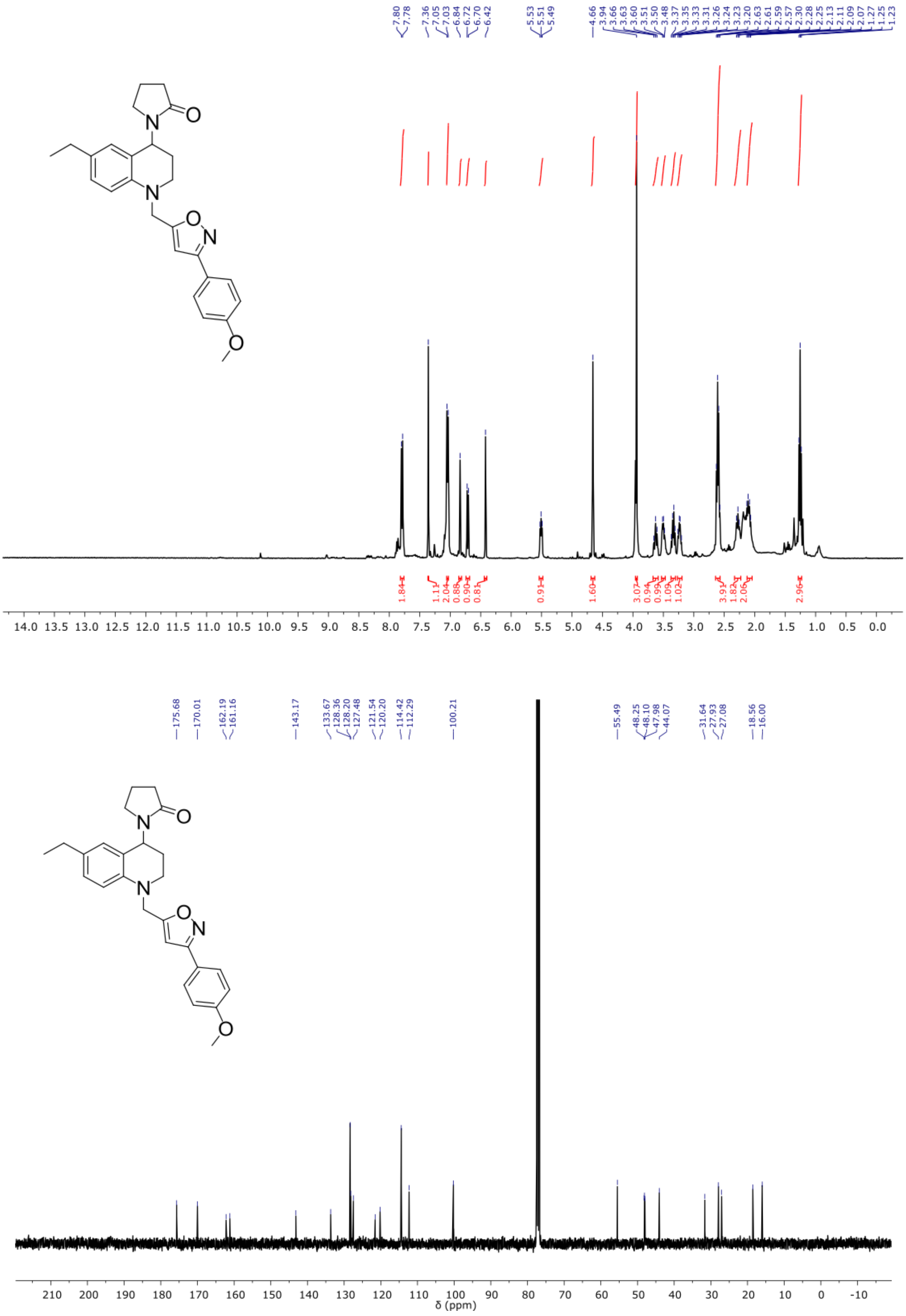

Figure S12. ^{1}H and ^{13}C-NMR spectra of 3-(4-methoxyphenyl)-5-((6′-ethyl-4′-(2′′-oxopyrrolidin-1′′-yl)-3′,4′-dihydroquinolin-1′(2′H)-yl)methyl)isoxazol **(6r).**

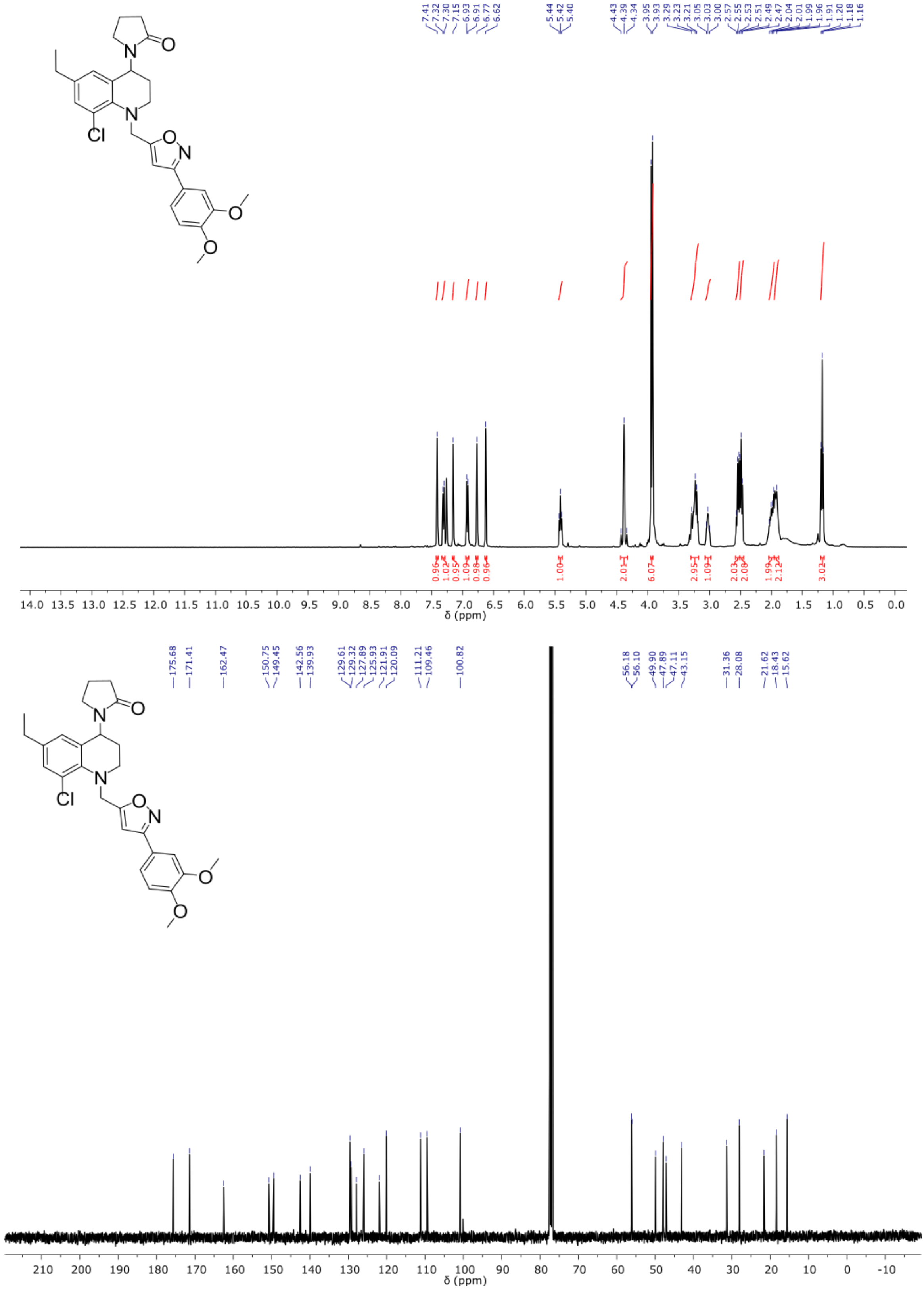

Figure S13. ^{1}H and ^{13}C-NMR spectra of 3-(3,4-dimethoxyphenyl)-5-((8′-chloro-6′-ethyl-4′-(2′′-oxopyrrolidin-1′′-yl)-3′,4′-dihydroquinolin-1′(2′H)-yl)methyl)isoxazol **(6s).**

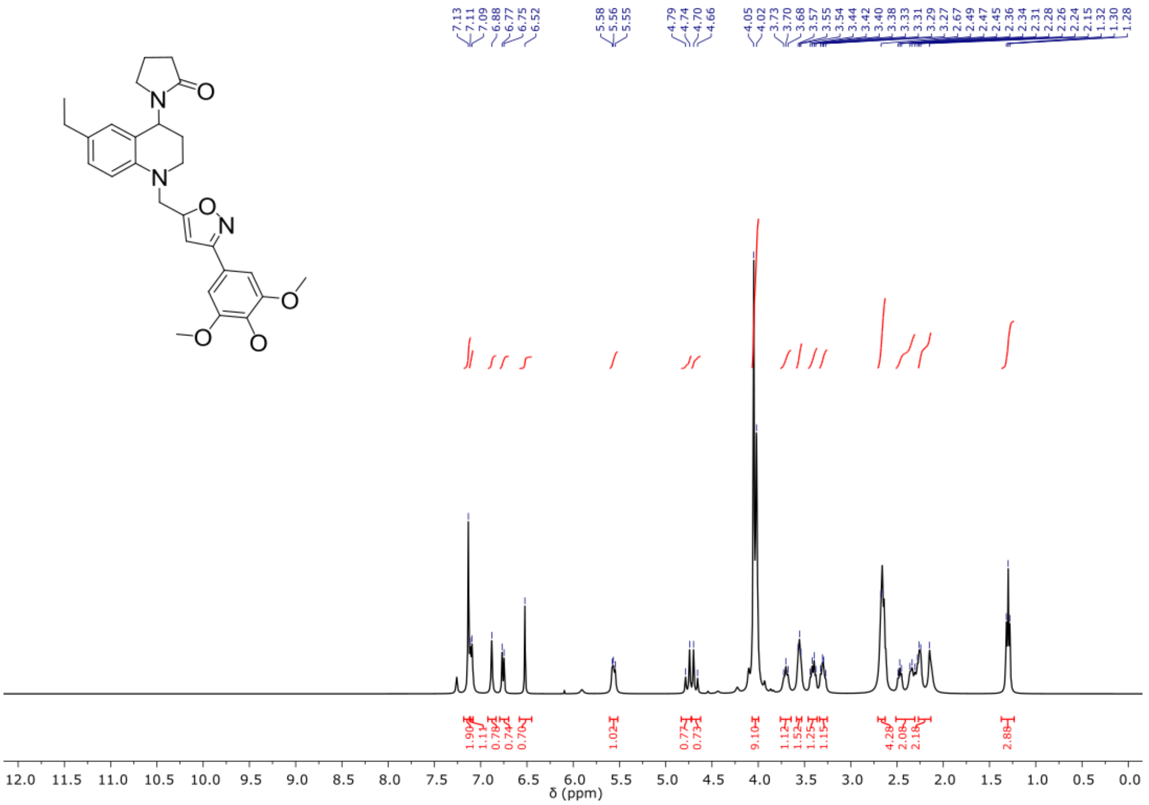

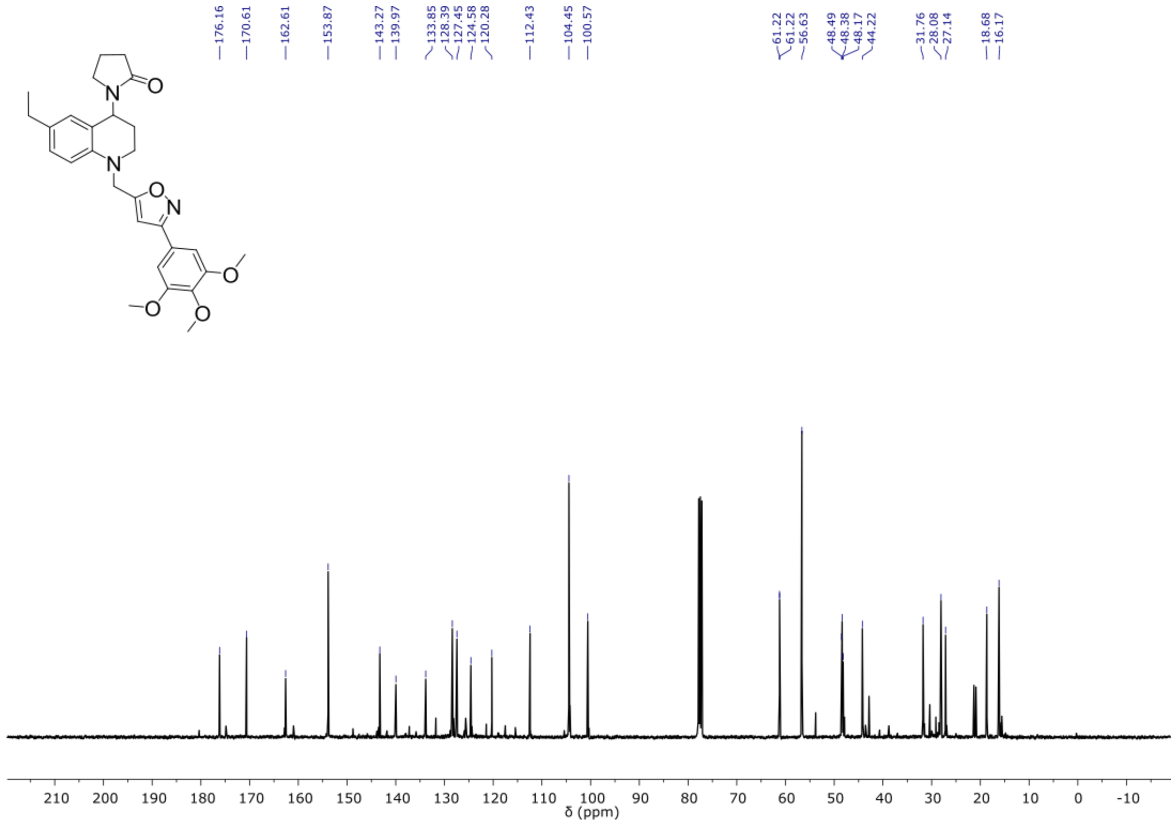

Figure S14. ^{1}H and ^{13}C-NMR spectra of 5-((6′-ethyl-4′-(2′′-oxopyrrolidin-1′′-yl)-3′,4′-dihydroquinolin-1′(2′H)-yl)methyl)-3-(3,4,5- trimethoxyphenyl)isoxazol **(6t).**

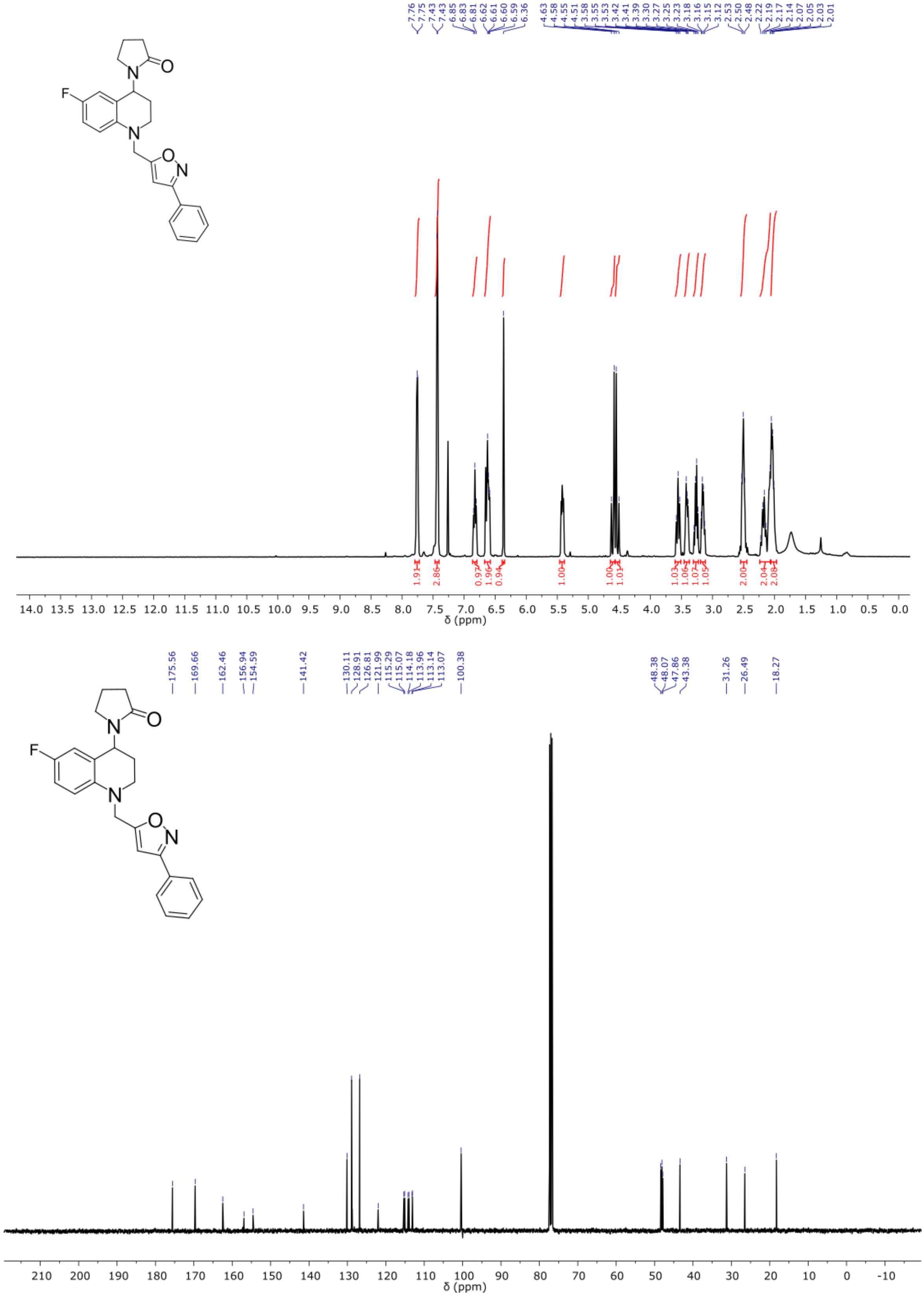

Figure S15. ^{1}H and ^{13}C-NMR spectra of 3-(phenyl)-5-((6′-fluoro-4′-(2′′-oxopyrrolidin-1′′-yl)-3′,4′-dihydroquinolin-1′(2′H)-yl)methyl)isoxazol **(6u).**

(6v)

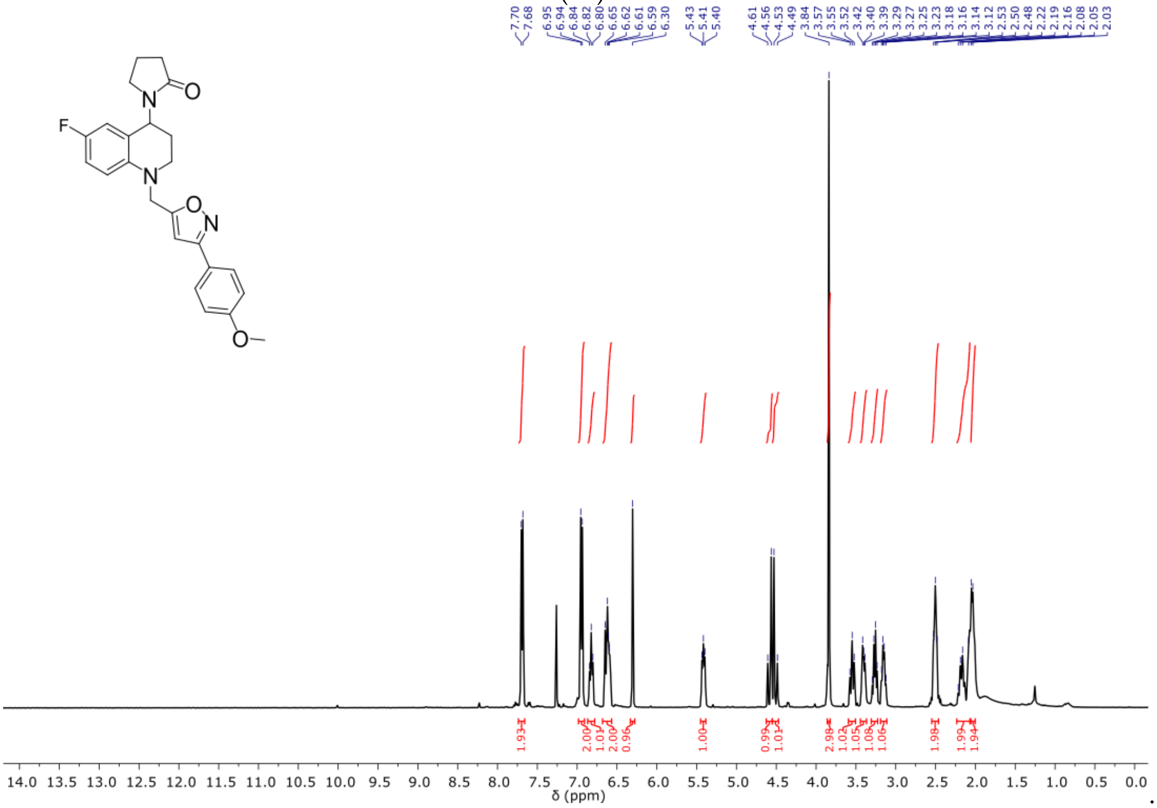

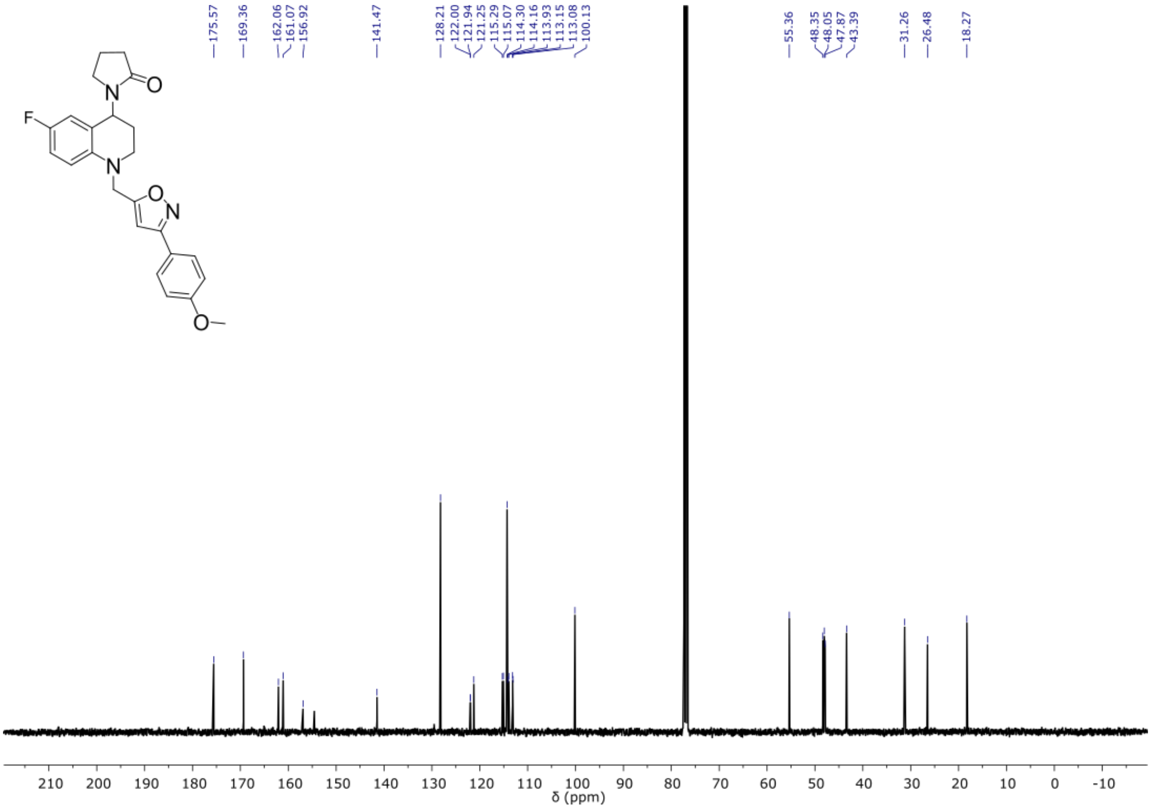

Figure S16. ^{1}H and ^{13}C-NMR spectra of 3-(4-methoxyphenyl)-5-((6′-fluoro-4′-(2′′-oxopyrrolidin-1′′-yl)-3′,4′-dihydroquinolin-1′(2′H)-yl)methyl)isoxazol.

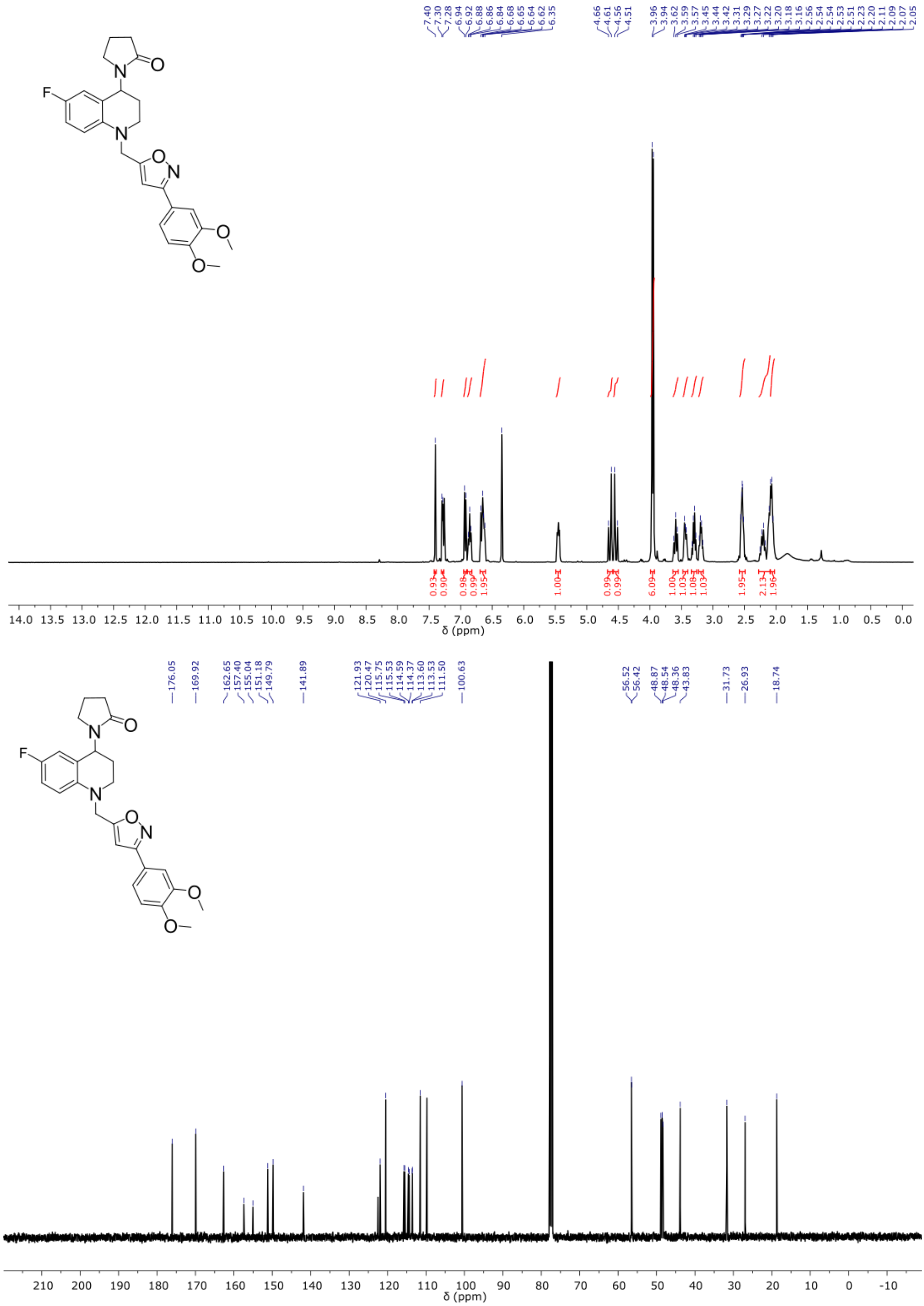

Figure S17. ¹H and ¹³C-NMR spectra of 3-(3,4-dimethoxyphenyl)-5-((6′-fluoro-4′-(2′′-oxopyrrolidin-1′′-yl)-3′,4′-dihydroquinolin-1′(2′H)-yl)methyl)isoxazol **(6w).**

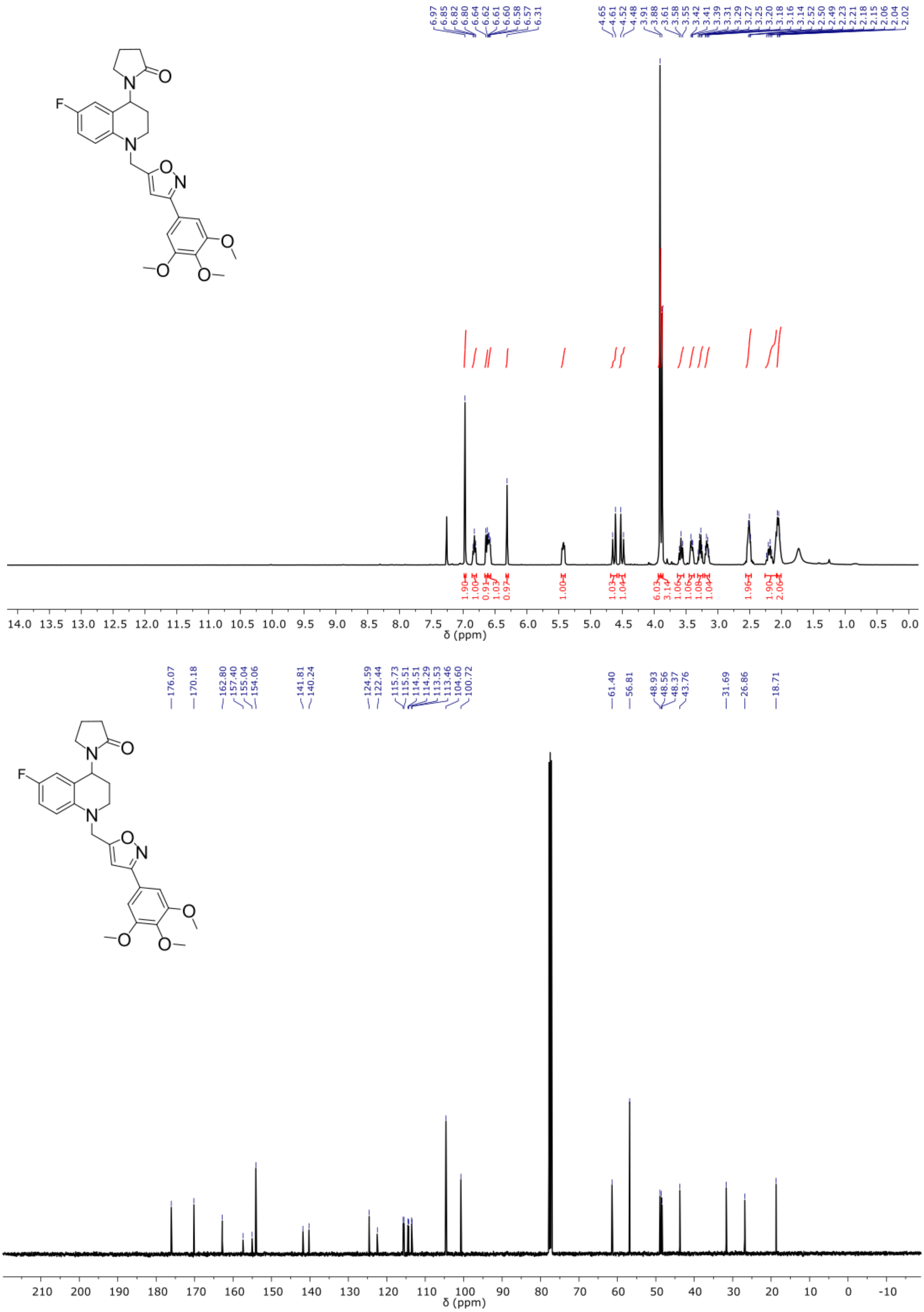

Figure S18. ^{1}H and ^{13}C-NMR spectra of 5-((6′-fluoro-4′-(2′′-oxopyrrolidin-1′′-yl)-3′,4′-dihydroquinolin-1′(2′H)-yl)methyl)-3-(3,4,5- trimethoxyphenyl)isoxazol **(6x).**

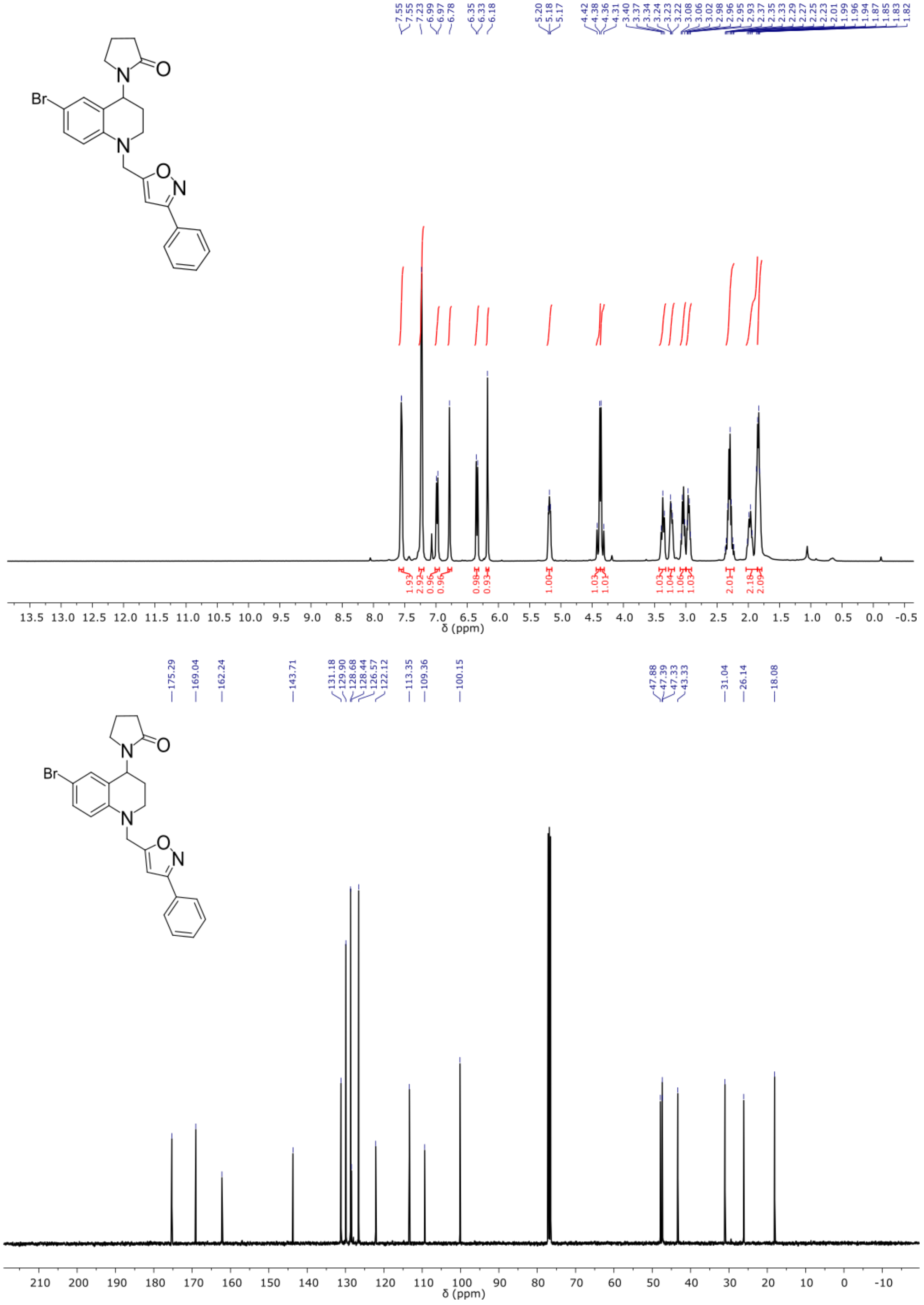

Figure S19. ^{1}H and ^{13}C-NMR spectra of 3-(phenyl)-5-((6′-bromo-4′-(2′′-oxopyrrolidin-1′′-yl)-3′,4′-dihydroquinolin-1′(2′H)-yl)methyl)isoxazol **(6y).**

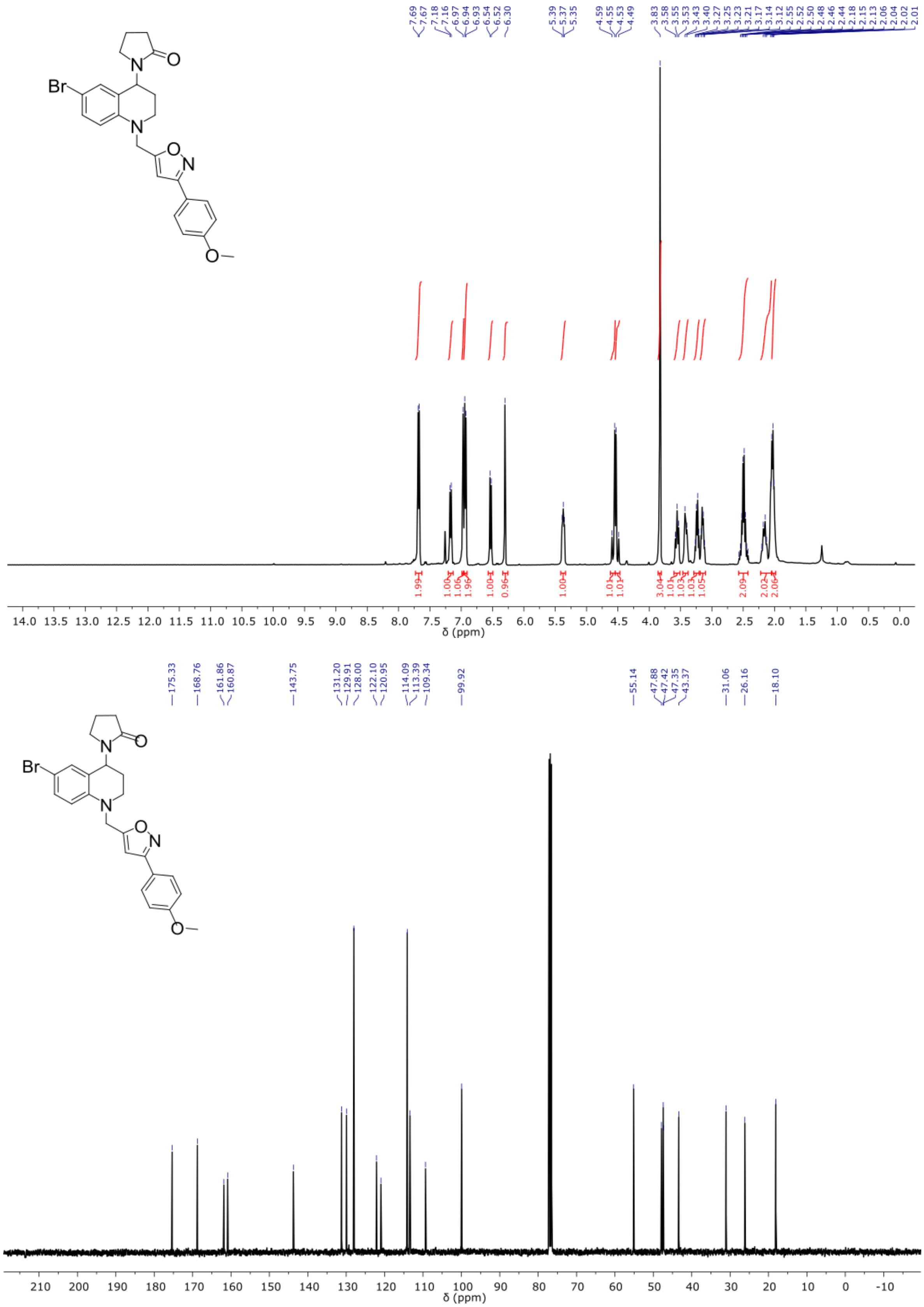

Figure S20. ^{1}H and ^{13}C-NMR spectra of 3-(4-methoxyphenyl)-5-((6′-bromo-4′-(2′′-oxopyrrolidin-1′′-yl)-3′,4′-dihydroquinolin-1′(2′H)-yl)methyl)isoxazol **(6z).**

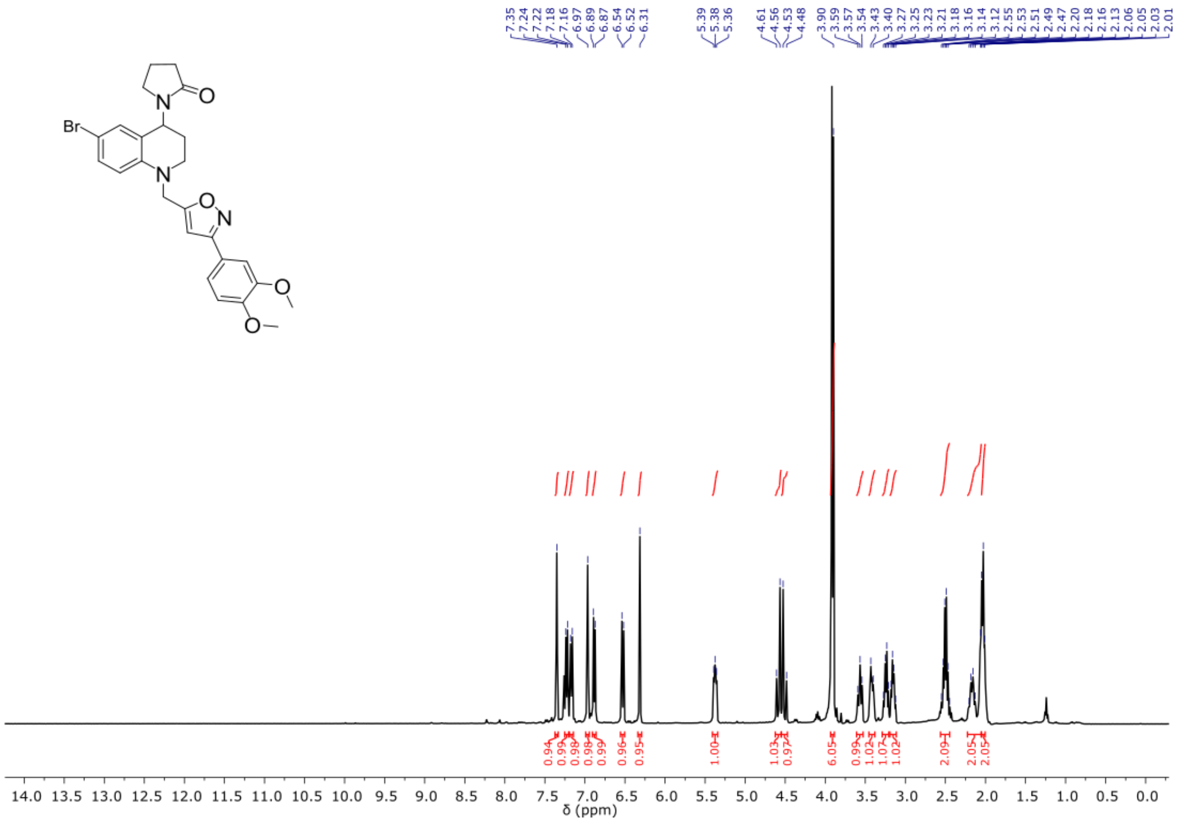

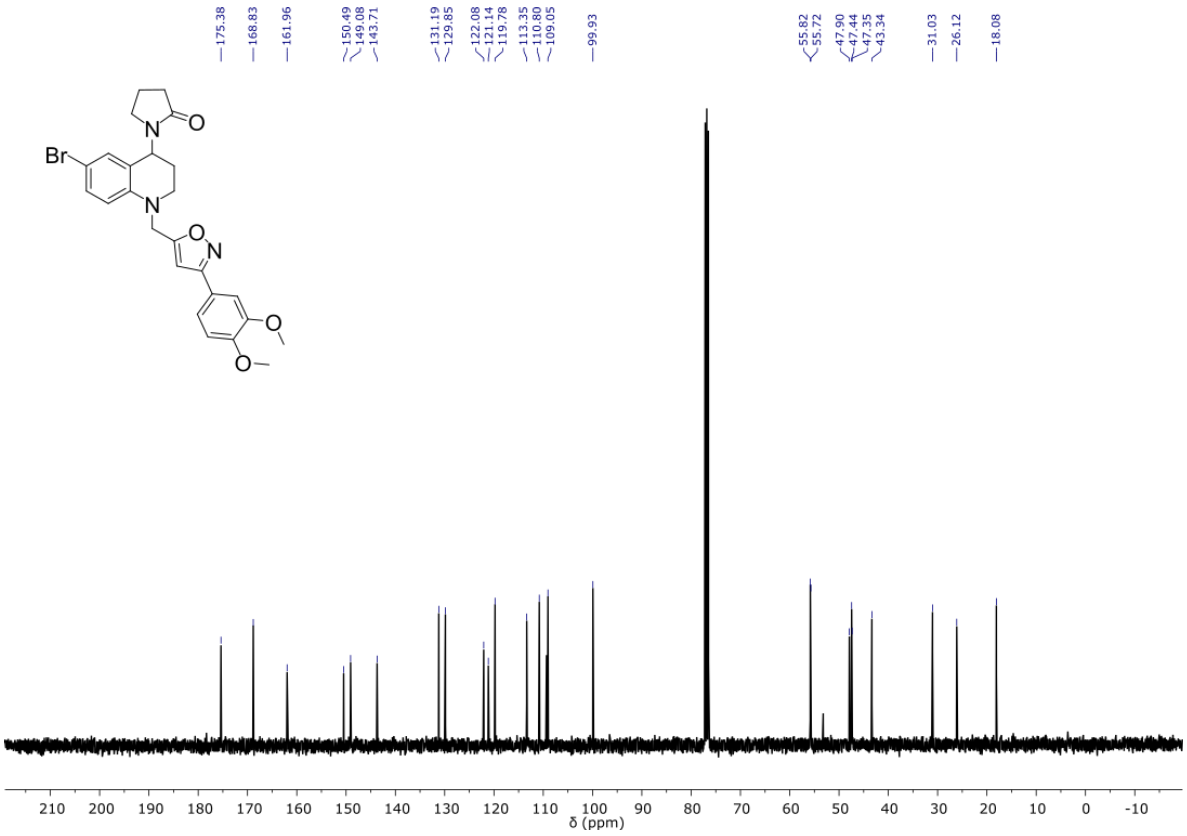

Figure S21. ^{1}H and ^{13}C-NMR spectra of 3-(3,4-dimethoxyphenyl)-5-((6′-bromo-4′-(2′′-oxopyrrolidin-1′′-yl)-3′,4′-dihydroquinolin-1′(2′H)-yl)methyl)isoxazol **(6aa).**

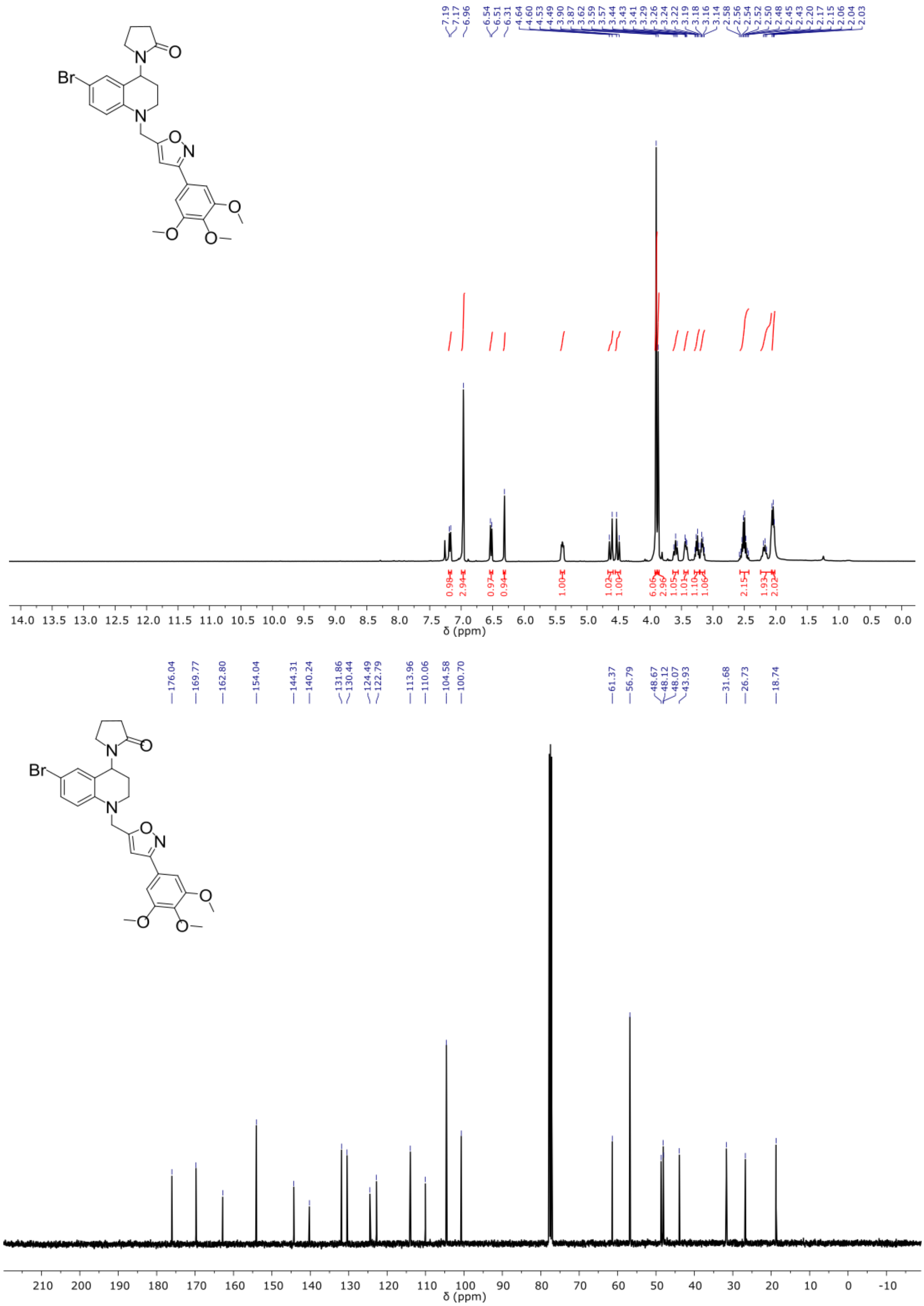

Figure S22. ^{1}H and ^{13}C-NMR spectra of 5-((6′-bromo-4′-(2′′-oxopyrrolidin-1′′-yl)-3′,4′-dihydroquinolin-1′(2′H)-yl)methyl)-3-(3,4,5- trimethoxyphenyl)isoxazol **(6ab).**

Table S1. Physicochemical parameters of new THQ – isoxazoline **5** molecular hybrids.

Comp 5	R_1	R_2	R_3	R_4	M.W. (g/mol)	Yield, %[a]	m.p ºC [b]

**a**	H	H	H	H	375.47	74	Yellow oil
**b**	H	H	OCH_3	H	405.49	64	Yellow oil
**c**	H	OCH_3	OCH_3	H	435.51	65	Yellow oil
**d**	H	OCH_3	OCH_3	OCH_3	465.54	65	Red oil
**e**	CH_3	H	H	H	389.49	65	Orange oil
**f**	CH_3	H	OCH_3	H	419.51	70	Orange oil
**g**	CH_3	OCH_3	OCH_3	H	449.54	63	Orange oil
**h**	CH_3	OCH_3	OCH_3	OCH_3	479.56	68	Orange oil
**i**	OCH_3	H	H	H	405.49	77	Red oil
**j**	OCH_3	H	OCH_3	H	435.51	70	Red oil
**k**	OCH_3	OCH_3	OCH_3	H	465.54	76	Red oil
**l**	OCH_3	OCH_3	OCH_3	OCH_3	495.56	68	Red oil
**m**	Cl	H	H	H	409.90	74	Yellow oil
**n**	Cl	H	OCH_3	H	439.93	64	Yellow oil
**o**	Cl	OCH_3	OCH_3	H	469.96	75	Yellow oil
**p**	Cl	OCH_3	OCH_3	OCH_3	499.98	75	Yellow oil

[a]Yields after column chromatography. [b]Uncorrected.

Table S2. Physicochemical parameters of new THQ – isoxazole **6** molecular hybrids.

Comp 6	R_1	R_2	R_3	R_4	R_5	M.W. (g/mol)	Yield, %[a]	m.p ℃ [b]
**a**	H	H	H	H	H	373.46	55	Orange oil
**b**	H	H	OCH_3	H	H	403.48	65	Red oil
**c**	H	OCH_3	OCH_3	OCH_3	H	463.53	75	Brown oil
**d**	CH_3	H	H	H	H	387.48	72	Red oil
**e**	CH_3	H	OCH_3	H	H	417.51	64	Red oil
**f**	CH_3	OCH_3	OCH_3	H	Cl	481.98	58	123 - 125
**g**	CH_3	OCH_3	OCH_3	OCH_3	H	477.56	63	Red oil
**h**	OCH_3	H	H	H	H	403.48	42	Brown oil
**i**	OCH_3	H	OCH_3	H	H	433.51	74	Red oil
**j**	OCH_3	OCH_3	OCH_3	H	Cl	497.98	86	128 - 130
**k**	OCH_3	OCH_3	OCH_3	OCH_3	H	493.56	74	Red oil
**l**	Cl	H	H	H	H	407.90	55	Red oil
**m**	Cl	H	OCH_3	H	H	437.92	51	Orange oil
**n**	Cl	OCH_3	OCH_3	H	H	467.95	68	Orange oil
**o**	Cl	OCH_3	OCH_3	H	Cl	502.39	88	157 - 159
**p**	Cl	OCH_3	OCH_3	OCH_3	H	497.98	85	Brown oil
**q**	CH_2CH_3	H	H	H	H	401.51	40	Brown oil
**r**	CH_2CH_3	H	OCH_3	H	H	431.53	60	Orange oil
**s**	CH_2CH_3	OCH_3	OCH_3	H	Cl	496.00	73	177 - 179
**t**	CH_2CH_3	OCH_3	OCH_3	OCH_3	H	491.58	72	Orange oil
**u**	F	H	H	H	H	391.44	89	Orange oil
**v**	F	H	OCH_3	H	H	421.47	77	Orange oil
**w**	F	OCH_3	OCH_3	H	H	451.49	75	Orange oil
**x**	F	OCH_3	OCH_3	OCH_3	H	481.52	87	Orange oil
**y**	Br	H	H	H	H	452.35	95	157 - 159
**z**	Br	H	OCH_3	H	H	482.37	67	102 - 104
**aa**	Br	OCH_3	OCH_3	H	H	512.40	70	Orange oil
**ab**	Br	OCH_3	OCH_3	OCH_3	H	542.43	43	Orange oil

[a]Yields after column chromatography. [b]Uncorrected.

Table S3. Main descriptors calculated for THQ-isoxazole/isoxazoline hybrid compounds using *QikProp* software.

Comp.	M.W. (g/mol)	log P (o/w)[a]	Mol. Vol (Å^3)[b]	Acceptor HB[c]	Donor HB[d]	PSA[e]	log S[f]	Oral Abs.[g]	5 Rule[h]
**5a**	375.469	3.276	1.208.799	6.700	0.000	57.198	-3.515	3	0
**5b**	405.496	3.158	1.268.532	7.450	0.000	61.602	-3.843	3	0
**5c**	435.522	3.293	1.349.289	8.200	0.000	64.632	-4.096	3	0
**5d**	465.548	3.349	1.426.988	8.950	0.000	76.062	-4.195	3	0
**5e**	389.496	3.430	1.254.086	6.700	0.000	53.323	-4.324	3	0
**5f**	419.522	3.483	1.328.231	7.450	0.000	61.604	-4.459	3	0
**5g**	449.549	3.507	1.398.204	8.200	0.000	69.936	-4.561	3	0
**5h**	479.575	3.555	1.464.587	8.950	0.000	74.160	-4.541	1	0
**5i**	405.496	3.128	1.264.505	7.450	0.000	61.667	-3.758	3	0
**5j**	435.522	3.181	1.338.652	8.200	0.000	69.951	-3.893	3	0
**5k**	465.548	3.308	1.421.193	8.950	0.000	74.320	-4.142	3	0
**5l**	495.574	3.373	1.494.725	9.700	0.000	84.095	-4.272	1	0
**5m**	409.914	3.607	1.237.977	6.700	0.000	53.323	-4.464	3	0

**5n**	439.941	3.661	1.312.201	7.450	0.000	61.604	-4.601	3	0
**5o**	469.967	3.796	1.392.932	8.200	0.000	64.607	-4.854	3	0
**5p**	499.993	3.797	1.462.131	8.950	0.000	75.053	-4.890	3	0
**6a**	373.454	3.769	1.201.928	5.500	0.000	53.616	-4.545	3	0
**6b**	403.480	3.821	1.275.970	6.250	0.000	61.908	-4.678	3	0
**6c**	463.532	3.675	1.400.995	7.750	0.000	76.366	-4.367	3	0
**6d**	387.480	4.093	1.261.257	5.500	0.000	53.616	-5.119	3	0
**6e**	417.507	4.145	1.335.297	6.250	0.000	61.908	-5.252	3	0
**6f**	481.978	4.510	1.415.180	7.000	0.000	66.600	-6.223	1	0
**6g**	477.559	4.233	1.487.517	7.750	0.000	75.914	-5.817	1	0
**6h**	403.480	3.798	1.272.946	6.250	0.000	61.931	-4.608	3	0
**6i**	433.506	3.851	1.347.103	7.000	0.000	70.215	-4.743	3	0
**6j**	497.977	4.209	1.425.486	7.750	0.000	75.076	-5.630	3	0
**6k**	493.558	3.867	1.485.677	8.500	0.000	83.084	-5.028	1	0
**6l**	407.899	4.272	1.245.984	5.500	0.000	53.624	-5.300	3	0
**6m**	437.925	4.325	1.320.141	6.250	0.000	61.908	-5.436	3	0
**6n**	467.951	4.454	1.402.857	7.000	0.000	66.355	-5.694	3	0
**6o**	502.396	4.786	1.411.422	7.000	0.000	62.915	-6.534	1	1
**6p**	497.977	4.120	1.519.308	9.250	0.000	75.066	-5.796	3	0
**6q**	401.507	4.433	1.314.576	5.500	0.000	53.616	-5.398	3	0
**6r**	431.533	4.485	1.388.631	6.250	0.000	61.908	-5.533	3	0
**6s**	496.005	4.881	1.472.201	7.000	0.000	66.596	-6.548	1	0
**6t**	491.586	4.448	1.517.144	7.750	0.000	76.257	-5.601	3	0
**6u**	391.444	4.004	1.217.486	5.500	0.000	53.616	-4.912	3	0
**6v**	421.470	4.056	1.291.644	6.250	0.000	61.901	-5.047	3	0
**6w**	451.496	4.183	1.374.149	7.000	0.000	66.329	-5.301	3	0
**6x**	481.523	3.849	1.490.513	9.250	0.000	75.069	-5.407	3	0
**6y**	452.350	4.343	1.254.193	5.500	0.000	53.781	-5.410	3	0
**6z**	482.376	4.399	1.328.457	6.250	0.000	61.908	-5.547	3	0

[a] log *P* for octanol/water (-2.0 - -6.5). [b]Total solvent accessible volume in cubic angstroms using a probe with a radius of 1.4 _A. [c] Estimated number of H-bonds that would be donated by the solute to water molecules in an aqueous solution. [d] Estimated number of H-bonds that would be accepted by solute from water molecules in an aqueous solution. [e] Van der Waals surface areas of polar nitrogen and oxygen atoms. [f] Predicted aqueous solubility, log *S*, *S* in mol dm^{-3}(-6.5 - 0.5). [g] Qualitative human oral absorption predicted: 1, 2 or 3 for low, medium or high.. [h] Violations number of Lipinski's of rule 5.
